# Concomitant Gradient Effects Across Field Strengths and Gradient Amplitudes: Improved Estimation of Errors and Correction of Concomitant Dephasing and Diffusion Weighting

**DOI:** 10.1002/mrm.70422

**Published:** 2026-06-11

**Authors:** Viktor Olsson, Felix Mortensen, Emil Ljungberg, Frederik Testud, Ronnie Wirestam, Malwina Molendowska, Filip Szczepankiewicz

**Affiliations:** ^1^ Department of Medical Radiation Physics Lund University Lund Sweden; ^2^ Siemens Healthcare AB Malmö Sweden

**Keywords:** asymmetric diffusion encoding, concomitant gradients, diffusion‐weighted magnetic resonance imaging, Maxwell terms

## Abstract

**Purpose:**

Deleterious effects from concomitant gradients are amplified at lower field strengths and stronger gradients. We aimed to show the signal errors caused by concomitant gradients across a wide range of hardware configurations and propose corrections that can be applied during experimental design and analysis.

**Theory and Methods:**

We derived a compact but accurate expression for concomitant gradients and simulated the associated signal bias across field strengths (0.03‐7 T) and gradient amplitudes (20‐600 mT/m) for asymmetric Maxwell‐compensated diffusion encoding gradient waveforms. Bias was assigned to “concomitant dephasing” or “concomitant diffusion weighting.” Concomitant dephasing was reduced by waveform correction that was deployed in the design stage. Concomitant diffusion weighting could be accounted for by using the actual gradient waveform during analysis.

**Results:**

Concomitant dephasing was caused by loss of Maxwell compensation during waveform resampling, with signal errors up to 100%, especially at low *B*_0_ and high *g*_max_. Our waveform correction yielded a categorical improvement to signal accuracy, reducing bias to < 1% for most systems. However, bias at extreme *B*_0_‐*g*_max_‐combinations remained > 1% (e.g., *B*_0_ = 3 T and *g*_max_ = 600 mT/m). Concomitant diffusion weighting caused biases above 1% at across all simulated MRI systems.

**Conclusion:**

Concomitant gradients have a relevant impact on signal accuracy, especially at low fields and ultra‐strong gradients. Concomitant dephasing must be considered for asymmetric waveforms, but can be largely suppressed by our correction method. Concomitant diffusion weighting is always present, regardless of waveform symmetry, but has a smaller impact and can be accounted for in the analysis.

## 1 Introduction

The recent introduction of human MRI systems with low magnetic field strengths or ultra‐strong gradients afford new opportunities for MRI as a clinical and research tool. However, the extended range of magnetic fields and gradients also introduces novel challenges in the design of MRI experiments. A major challenge stems from the increased impact of concomitant magnetic fields. These are unintended contributions to the magnetic field, or field gradients, as described by Maxwell's equations [1] and are therefore often referred to as “concomitant gradients” or “Maxwell terms.”

The effects of Maxwell terms increase with decreasing magnetic field and with increasing gradient amplitude. These effects manifest in a number of different ways, including image artifacts [2, 3, 4, 5], errors related to spin phase and flow encoding [6, 7], and diffusion encoding [8, 9, 10]. In most clinical diffusion MRI (dMRI) applications, the diffusion encoding gradients are arranged symmetrically around the refocusing radiofrequency (RF) pulse in a spin echo sequence. This symmetry effectively prevents gross signal errors (concomitant dephasing), and for field strengths of approximately 1.5‐3 T and gradient amplitudes up to 80 mT/m, other effects (concomitant diffusion weighting) are small or negligible. Gradient waveforms that are asymmetric, that is, not arranged symmetrically around the refocusing pulse, are more sensitive to Maxwell terms [3, 8]. Despite this drawback, asymmetric gradient waveforms are vastly more flexible and enable reduced eddy current effects [11, 12, 13, 14], single‐shot isotropic diffusion encoding [15, 16, 17, 18], and improved encoding efficiency in waveforms designed to provide conventional or advanced diffusion contrasts [19, 20, 21, 22].

A method for “Maxwell compensation,” wherein the asymmetric diffusion encoding gradient waveforms were designed to minimize concomitant gradient effects while maximizing encoding efficiency, has previously been proposed [8]. However, that method was developed to be applicable to MRI systems with main magnetic field strengths *B*_0_ ≈ 3 T and maximal gradient amplitudes *g*_max_ ≈ 80 mT/m, i.e., hardware parameters that were representative of clinical scanners with relatively high performance at that time. The increasing availability of portable ultra‐low‐field systems (*B*_0_ < 0.1 T) [23, 24, 25], stationary low‐field systems (*B*_0_ < 1 T) [26], and systems with ultra‐strong gradients (*g*_max_ ≥ 200 mT/m) [27, 28, 29, 30, 31] has expanded the relevant parameter space, warranting a reevaluation of the previously proposed approach to verify its validity and generalizability.

In this work, we explored how the wider parameter space (in terms of main magnetic field and gradient strength) challenges three specific points in the design of asymmetric diffusion encoding gradients: (*i*) the validity of Maxwell compensation based on a second‐order approximation of concomitant gradients, (*ii*) the effect of concomitant dephasing caused by interpolation of Maxwell‐compensated gradient waveforms, and (*iii*) the contributions of concomitant gradients to the diffusion weighting. These three factors were investigated in terms of signal bias across a wide range of MRI hardware configurations, and methods to improve or circumvent potential signal biases were developed.

## 2 Theory

In this section, a brief description of concomitant gradients is given, but the reader is also referred to detailed works [6, 8, 9, 10, 32, 33]. Throughout, scalars are denoted by italicized letters, and vectors as well as tensors are written in boldface letters. It is assumed that the main magnetic field vector is directed along the z‐direction, such that $B_{0}={\left[0\;\;0\;\;B_{0}\right]}^{T}$, and all gradient waveforms $g\left(t\right)={\left[g_{x}\left(t\right)\;g_{y}\left(t\right)\;g_{z}\left(t\right)\right]}^{T}$ are stated as the physical gradients, not including the effect of refocusing pulses or non‐homogeneity caused by gradient coil design. Finally, where appropriate, the desired, actual, and concomitant variants of given quantities are distinguished by subscripts “d,” “a,” and “c,” respectively.

### 2.1 Concomitant Magnetic Fields and Gradients

As a consequence of the laws described by the Maxwell‐Heaviside equations, perfectly linear spatial variations in a magnetic field are not possible since they would introduce a non‐zero magnetic field curl and divergence, and these must be zero in the absence of electrical currents [1]. This means that gradient fields that are parallel to **B**_0_, i.e., those generally considered in MRI, are always accompanied by *concomitant* gradients. These contribute to “magnetic field non‐linearity” and “gradient non‐uniformity.”^1^

**FIGURE 1:**
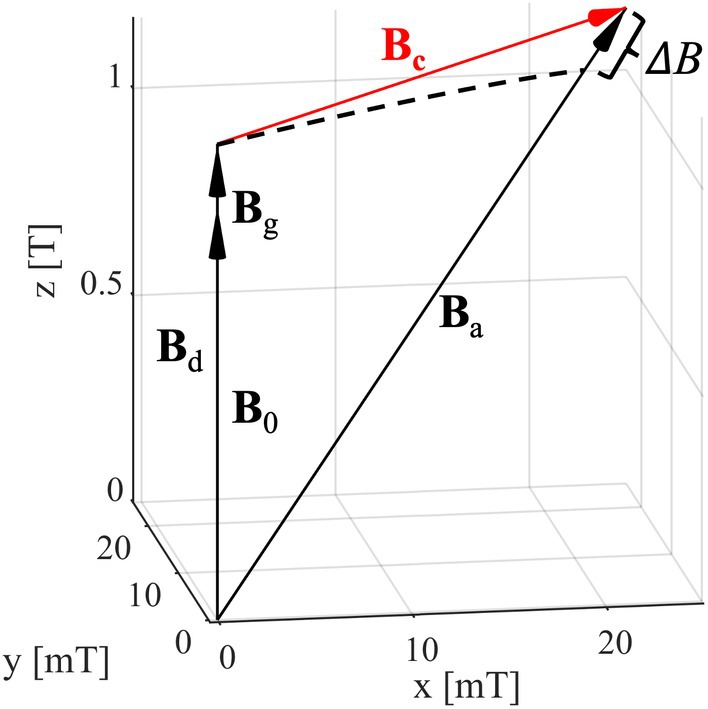
The concomitant magnetic field vector (**B**_c_) is orthogonal to the desired magnetic field (**B**_d_ = **B**_0_ + **B**_g_) and causes an undesired change of the local spin precession frequency ($\Delta B=\left|B_{a}\right|-\left|B_{d}\right|$). Dephasing occurs when the concomitant gradient (**g**_c_) violates the spin echo condition. Moreover, if **g**_c_ fulfills the spin echo condition, the actual gradient (**g**_a_) will nevertheless deviate from the desired gradient (**g**_d_), resulting in a non‐zero concomitant diffusion weighting.

The actual magnetic field vector can be calculated as a function of the desired gradient and the position vector $r={\left[x\;y\;z\right]}^{T}$, according to [6]

(1) $$B_{a}\left(r\right)\approx \underset{B_{d}}{\underbrace{\left[\begin{array}{c} 0\\ 0\\ B_{0} \end{array}\right]+\left[\begin{array}{ccc} 0 & 0 & 0\\ 0 & 0 & 0\\ g_{d,x} & g_{d,y} & g_{d,z} \end{array}\right]\cdot r}}+\underset{B_{c}}{\underbrace{\left[\begin{array}{ccc} -\;\alpha g_{d,z} & \beta & g_{d,x}\\ \beta & \left(\alpha -1\right)g_{d,z} & g_{d,y}\\ 0 & 0 & 0 \end{array}\right]\cdot r}},$$

where “·” denotes the inner product, α is the coil symmetry factor (unit of 1), and β is a coil factor (in units of T/m). Note that the desired magnetic field vector, **B**_d_, is intended to be parallel with **B**_0_, but that the actual magnetic field vector, **B**_a_, has a magnitude and direction that depends on concomitant effects (Figure 1). The undesired concomitant contribution to the magnetic field magnitude can be written as $\Delta B=\left|B_{a}\right|-\left|B_{d}\right|$.

By substituting appropriate rows in Equation (1) by $v=\left[-\alpha g_{d,z}\;\beta \;g_{d,x}\right]$, and $w=\left[\beta \;\left(1-\alpha \right)g_{d,z}\;g_{d,y}\right]$, the following is obtained

(2) $$\begin{array}{c} B_{a}\left(r\right)\approx \end{array}\left[\begin{array}{l} 0\\ 0\\ B_{0} \end{array}\right]+\left[\begin{array}{l} v\\ w\\ g_{d} \end{array}\right]\cdot r.$$

To express concomitant effects in terms of the gradient rather than the magnetic field, the actual gradient can be calculated as the spatial derivative of the magnitude of the actual magnetic field vector, $\left|B_{a}(r)\right|$, according to

(3) $$g_{a}\left(r\right)=\frac{\partial \left|B_{a}\left(r\right)\right|}{\partial r}=\underset{g_{d}}{\underbrace{\frac{\partial \left|B_{d}\right|}{\partial r}}}+\underset{g_{c}\left(r\right)}{\underbrace{\frac{\partial \Delta B\left(r\right)}{\partial r}}}.$$

An approximate expression for **g**_a_ is often derived from a truncated Taylor expansion of the actual field [6]. However, here we propose to avoid this approximation by deriving **g**_a_ including all higher‐order effects, as described in the Supporting Information. By doing so, the following compact expression is obtained

(4) $$g_{a}=\frac{g_{d}B_{d}+\left(v^{⊗2}+w^{⊗2}\right)\cdot r}{\sqrt{B_{d}^{2}+{\left(v\cdot r\right)}^{2}+{\left(w\cdot r\right)}^{2}}},$$

where ⊗ is the outer product, and the desired magnetic field strength is $B_{d}=\left|B_{d}\right|=B_{0}+g_{d}\cdot r$. Finally, the concomitant gradient can be isolated as the difference between the desired and actual gradient waveforms

(5) $$\begin{array}{r} g_{c}\left(t,r\right)=g_{a}\left(t,r\right)-g_{d}\left(t\right). \end{array}$$

Note that the concomitant gradient instantly assumes its value as the desired gradient waveform is modulated in time [1]. Moreover, it is helpful to note that concomitant gradients, to leading order, scale as follows [6]

(6) $$\left|g_{c}\right|\propto \frac{{\left|g_{d}\right|}^{2}\cdot \left|r\right|}{B_{0}}.$$

Hence, we anticipate more pronounced concomitant gradient effects at low magnetic field strengths, higher gradient amplitudes, and at longer distances from the isocenter.

### 2.2 Signal Bias due to “Concomitant Dephasing”

In the context of dMRI with asymmetric gradient waveforms, the primary source of signal error is the undesired spin dephasing from “non‐balanced” concomitant gradients [1, 8, 9]. A balanced gradient waveform fulfills the spin echo condition (**k** ≈ 0), i.e., it has a negligible dephasing vector at the end of the encoding period

(7) $$k=\gamma \int_{0}^{\tau }g\left(t\right)h\left(t\right)dt,$$

where *τ* is the echo time and *h*(*t*) = ±1 is a sign function with opposite signs in each time interval separated by a refocusing pulse. If the desired gradient waveform fulfills the spin echo condition (**k**_d_ = 0), only the concomitant gradient (**g**_c_, Equation 5) will contribute to the phase difference between two nearby spins at positions **r**_0_ and **r**_0_ + Δ**r** according to

(8) $$\begin{array}{rr} \Delta \phi_{c} & =\gamma \int_{0}^{\tau }g_{a}\left(t,r_{0}\right)\cdot \Delta r\;h\left(t\right)\;dt\;\underset{\text{if}\;k_{d}=0}{\underbrace{=}}\;\gamma \int_{0}^{\tau }g_{c}\left(t,r_{0}\right)\cdot \Delta r\;h\left(t\right)\;dt\\ & =k_{c}\left(r_{0}\right)\cdot \Delta r, \end{array}$$

where **k**_c_ is the concomitant dephasing vector. Thus, a non‐zero **k**_c_ will cause a linear phase dispersion around the point **r**_0_. Following the formalism by Baron et al. [9], the dephasing in a voxel that is homogeneously populated by spins will cause a signal attenuation factor (*AF*_ϕ_) that can be approximated by a sinc function, according to

(9) $$AF_{\phi }\approx \left|\text{sinc}\left(\frac{u_{s}\cdot k_{c}\left(r_{0}\right)}{2\pi }\right)\right|,$$

where **u**_s_ is a vector that is normal to the slice plane and has a magnitude equal to the slice thickness. Thus, the inner product **u**_s_ *·* **k**_c_ captures the dephasing moment in the through‐slice direction. The relative signal bias due to dephasing (*SB*_ϕ_) can then be defined as

(10) $${\text{SB}}_{\phi }=AF_{\phi }-1.$$

Note that this only includes the signal lost due to through‐slice dephasing, which is often the dominant, but not the only, contribution to the bias [8, 9]. Thus, actual measurements are expected to exhibit somewhat larger biases. For a given waveform that is kept at a constant nominal b‐value by scaling its duration, *SB*_ϕ_ scales approximately as

(11) $${\text{SB}}_{\phi }\propto {\left|k_{c}\right|}^{2}\propto \frac{{\left|g_{d}\right|}^{8⁄3}}{B_{0}^{2}}.$$

### 2.3 Signal Bias due to “Concomitant Diffusion Weighting”

Since the concomitant gradient alters the diffusion encoding gradient waveform, it will always affect the diffusion weighting. Although there are several studies on concomitant gradient effects [6, 8, 9, 10], they have, to the best of our knowledge, not addressed the effect of concomitant gradients on the diffusion weighting itself. Indeed, since **g**_c_ is small compared to **g**_d_, its impact in terms of diffusion weighting has been either overlooked or assumed to be vanishingly small. Nevertheless, the wide range of hardware configurations, in addition to the fact that concomitant diffusion weighting exists regardless of waveform symmetry, warrants an evaluation of their impact on the diffusion‐weighted signal and the b‐value.

The actual diffusion encoding b‐tensor (**b**, a 2^nd^ order tensor with rank(**b**) ≤ 3) is defined as the outer product of the actual dephasing vector with itself [34], according to

(12) $$b_{a}\left(r\right)=\int_{0}^{\tau }q_{a}\left(t,r\right)⊗q_{a}\left(t,r\right)dt,$$

where $q_{a}\left(t,r\right)$ is defined as^2^

(13) $$q_{a}\left(t,r\right)=\gamma \int_{0}^{t}g_{a}\left(t^{\prime },r\right)h\left(t^{\prime }\right)\;dt^{\prime }=\gamma \int_{0}^{t}\left(g_{d}\left(t^{\prime }\right)+g_{c}\left(t^{\prime },r\right)\right)h\left(t^{\prime }\right)\;dt^{\prime }.$$

By omitting or including **g**_c_ in Equations (12) and (13), the desired or actual b‐tensors (**b**_d_ and **b**_a_) can be calculated at any position **r**. The corresponding b‐values can then be calculated as the trace of the corresponding b‐tensor, b=Tr(b). The actual b‐tensor may differ from the desired b‐tensor in terms of size, shape, and rotation, although the latter two are not investigated in this work. Regardless, these deviations from the desired diffusion weighting can be accounted for by using the actual b‐tensor in the analysis (Equation 12).

The error in b‐value may be translated to a more tangible signal error by evaluating it in a simple, but representative, model system. Here, an isotropic and exponential signal decay is used to obtain the signal attenuation factor

(14) $$AF_{b}=\exp\left(-\left(b_{a}-b_{d}\right)D\right),$$

where *D* is the diffusivity, and the concomitant diffusion weighting strength is $b_{c}=b_{a}-b_{d}$. The signal bias due to inaccurate diffusion weighting (*SB*_b_), in analogy with Equation (10), becomes

(15) $${\mathrm{SB}}_{b}=AF_{b}-1.$$

Finally, for a waveform scaled in time to maintain constant nominal b‐value, *SB*_b_ can be shown to scale as

(16) $${\mathrm{SB}}_{b}\propto b_{c}\propto \frac{\left|g_{d}\right|}{B_{0}}.$$

## 3 Methods

Systematic errors due to two distinct mechanisms, concomitant dephasing and concomitant diffusion weighting, were investigated across multiple possible MRI system configurations to cover a wide range of challenging parameter combinations. “Maxwell‐compensated” diffusion encoding gradient waveforms were tailored to each MRI system, and the effects of concomitant gradients were estimated using the framework in the theory. To suppress errors from concomitant dephasing after gradient waveform interpolation, a correction algorithm was proposed and demonstrated. Throughout, errors below an arbitrary signal error threshold of 1% were considered to be negligible [8]. Importantly, this work focuses on concomitant gradient effects from the diffusion encoding gradients, i.e., excluding the potential effects from imaging gradients [10, 32].

### 3.1 MRI Hardware Configurations and Imaging Parameters

To consider currently existing MRI systems as well as hardware configurations that may emerge in the foreseeable future, main magnetic fields *B*_0_ ranging from 0.03 to 7 T and maximum gradient amplitudes (*g*_max_) ranging from 20 to 600 mT/m were considered. For simplicity, the rise time for each system was fixed at 1 ms, meaning the gradient amplitude reaches *g*_max_ in 1 ms. Furthermore, in Equation (1), we assumed symmetric gradient coils with α = ½ and β = 0 [6, 10], although Maxwell compensation generalizes to arbitrary gradient coil asymmetry [35] as shown in the Supporting Information. Additionally, four systems (A‐D) were highlighted to represent (A) a low‐field scanner (*B*_0_ = 0.2 T with *g*_max_ = 40 mT/m), (B and C) conventional clinical scanners (*B*_0_ = 1.5 and 3 T with *g*_max_ = 40 and 80 mT/m), and (D) a scanner with ultra‐strong gradients (*B*_0_ = 3 T with *g*_max_ = 300 mT/m).

Since the effects of concomitant gradients depend on several imaging parameters [9], comparability across systems was maintained by using a fixed set of imaging parameters. A slice‐selective spin echo with 2D EPI readout and a slice thickness of 5 mm was assumed, and the field‐of‐view (FOV) was defined as a sphere with a radius of 10 cm centered at the gradient system isocenter. To account for the asymmetric encoding times before and after the refocusing pulse (δ_1_ and δ_2_), the second encoding period was assumed to be 6 ms shorter than the first, and the refocusing block was assumed to have a duration of 8 ms, inspired by realistic parameters used at multiple MRI systems by Szczepankiewicz et al. [36].

### 3.2 Numerical Optimization of Maxwell‐Compensated Gradient Waveforms

Diffusion encoding gradient waveform optimization was performed with “Maxwell compensation” [8] using numerical optimization [19] to maximize encoding efficiency, i.e., to achieve maximal b‐value for a given encoding time configuration. Briefly, Maxwell compensation imposes a constraint during gradient waveform optimization that ensures that the actual gradient waveform is balanced, that is, **k**_a_ ≈ 0 (Equation 7), for all rotations and relevant gradient amplitude scaling factors of the waveform. This is achieved by constraining the so‐called Maxwell index, defined as

(17) $$\mu =\frac{\gamma }{B_{0}}{\left|\int_{0}^{\tau }\;g_{d}\left(t\right)⊗g_{d}\left(t\right)\;h\left(t\right)dt\right|}_{\text{Frob}}\approx \frac{\gamma }{B_{0}}{\left|\sum_{i=1}^{n}g_{d,i}⊗g_{d,i}h_{i}∆t\right|}_{\text{Frob}},$$

where the right‐hand side is the discrete form implemented in the numerical waveform optimization, *n* is the number of control points on each axis, and Δ*t* is the temporal resolution. Note that μ is closely related to the Maxwell index from our previous work [8], although the current formalism extends it to accommodate the effects of the targeted spin species (γ) and the magnetic field strength of the MRI system (*B*_0_). These extensions enabled us to use a single threshold for μ across all system configurations. Importantly, μ only includes the contributions of concomitant gradient effects up to order *g* ^2^/*B*_0_. Therefore, it does not explicitly suppress the contributions from higher order effects.

For all MRI hardware combinations, a Maxwell index threshold of μ < μ_thr_ = 270 m^−2^ was used, based on the study by Szczepankiewicz et al. [8]. The waveforms were optimized to yield a b‐value of approximately 2 ms/μm^2^ with spherical b‐tensor encoding. Spherical b‐tensor encoding was employed because it is usually the most challenging waveform design in terms of optimization complexity and scanner demands [22], and all results generalize across arbitrary shapes of the b‐tensor. The waveforms were optimized at a temporal resolution of 1 ms to reduce the computational complexity, and the *l* ^2^‐norm of each waveform was limited to *g*_max_ [19]. Finally, the gradient waveforms were resampled to a temporal resolution of 10 μs by linear interpolation to conform with typical raster times used at clinical MRI systems.

### 3.3 Gradient Waveform Correction to Recover Maxwell Compensation After Interpolation

Maxwell‐compensated gradient waveforms are optimized at a coarse temporal resolution due to computational constraints [8, 19]. This means that, at the time of optimization, low‐resolution waveforms have a Maxwell index that complies with the threshold (Equation 17). However, when waveforms are interpolated (e.g., Δ*t* = 1 ms → 10 μs), the Maxwell index is not preserved and the compensation can fail. Thus, the process of interpolation can induce concomitant dephasing [8].

Based on the intuition that linear interpolation yields waveforms that are close to being Maxwell compensated, we expect that only minor adjustments are necessary to restore Maxwell compensation. To this end, we developed a method for fine‐tuning interpolated gradient waveforms to achieve negligible concomitant dephasing (μ < μ_thr_ and **k**_a_ ≈ 0 for all relevant **r**) while maintaining the overall shape of the original gradient waveform.

**FIGURE 2:**
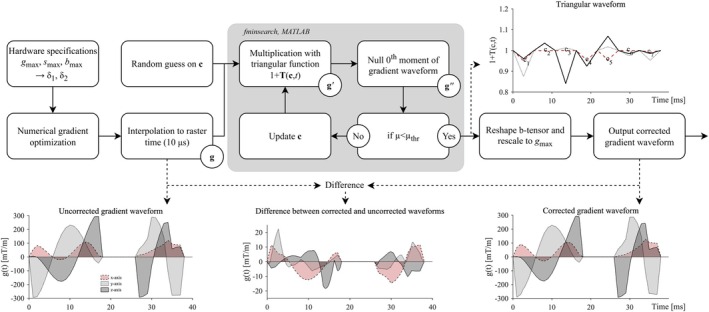
Schematic description of the correction algorithm. The flowchart should be interpreted from the left upper corner to the right lower corner. First, the specifications for the hardware and the gradient waveform need to be stated, and the time resolution of the waveform should correspond to the raster time of the scanner. The correction algorithm, which is an iterative loop that returns a Maxwell‐corrected gradient waveform, can then be applied. The *l* ^2^‐norm of the waveform is rescaled to *g*_max_, and the b‐tensor is reshaped to be spherical before the corrected waveform is returned. The time courses of the gradient waveforms designed with *B*_0_ = 3 T and *g*_max_ = 300 mT/m at different steps of the algorithm are presented in the bottom row. The waveform in the middle is the difference between the waveform before and after correction. The waveform before correction suffers from concomitant dephasing, while the waveform after correction has a negligible residual moment. Finally, the triangular waveform used for correction in this case is shown on the top right.

The correction algorithm (Figure 2) employs iterative minimization (*fminsearch*, MATLAB 2024b, The MathWorks, Natick, MA, USA), which by default uses the Nelder‐Mead simplex direct search method, to find a perturbation of the gradient waveform that reduces the Maxwell index below a user‐defined threshold value while maintaining the spin echo condition (Equation 7). As shown in Figure 2, each iteration performs two modifications to the waveform. First, the gradient waveform is multiplied by a triangular waveform, 1 + **T**(**c**,*t*). The triangular waveform comprises *n* equally spaced isosceles triangles per axis, where the set of triangles is described by their amplitudes, **c** = [*c*_1_ *c*_2_ … *c*_*n*·3_]. It is the vector **c**, with *n*·3 degrees of freedom, that is optimized to achieve the desired Maxwell index. The initial guess for all elements in **c** is random and drawn from a uniform distribution between −0.05 and 0.05 to stay close to zero. This step creates an intermediate gradient waveform

(18) $$\begin{array}{r} g_{d}^{\prime }\left(t\right)=g_{d}\left(t\right)⊙\left(1+T\left(c,t\right)\right), \end{array}$$

where ⊙ denotes element‐wise multiplication (Hadamard product). Second, the intermediate waveform is balanced by calculating its 0^th^ moment, $k_{d}^{\prime }$ (Equation 7). If it is not zero, the parts of the waveform that can be *reduced* to null the dephasing vector are identified. The parts of the waveform that show this characteristic are those that have the same sign as the dephasing vector on each axis, as captured by the binary mask

(19) $$\begin{array}{r} s\left(t\right)=\left\{\begin{array}{l} 1\;\text{if}\;g_{d}^{\prime }\left(t\right)⊙k_{d}^{\prime }\geq 0\;\left(\text{same}\;\mathrm{sign}\right)\\ 0\;\text{if}\;g_{d}^{\prime }\left(t\right)⊙k_{d}^{\prime }<0\;\left(\text{opposite}\;\mathrm{sign}\right). \end{array}\right. \end{array}$$

To obtain the factor by which the sub‐selected parts should be reduced, the 0^th^ moment of $g_{d}^{\prime }\left(t\right)$, including only parts where **s**(*t*) = 1, meaning the dephasing vector of the masked **g**_d_, is calculated, such that

(20) $$\begin{array}{r} k_{s}=\gamma \int_{0}^{\tau }s\left(t\right)⊙g_{d}^{\prime }\left(t\right)dt, \end{array}$$

resulting in the multiplication factor for each axis being the element‐wise division

(21) $$f=k_{d}^{\prime }⁄k_{s}.$$

The Maxwell‐corrected, interpolated, and balanced waveform can then be written as

(22) $$\begin{array}{r} g_{d}^{\prime \prime }\left(t\right)= \end{array}g_{d}^{\prime }\left(t\right)⊙\left(1-s\left(t\right)⊙f\right).$$

After each iteration, the Maxwell index of $g_{d}^{\prime \prime }\left(t\right)$ is calculated and compared to the threshold value (μ_thr_). The process is terminated if the current Maxwell index is below the threshold. To facilitate a fair comparison between waveforms before and after correction, both the maximal gradient amplitude and the shape of the b‐tensor of the corrected waveform are adjusted to be the same as before the correction, as described by Szczepankiewicz et al. [22].^3^ This introduces a slight re‐scaling of **g**(*t*) and therefore preserves the Maxwell compensation [8].

### 3.4 Simulation of Signal Bias due to Concomitant Dephasing

To evaluate the efficacy of the Maxwell compensation across a wide range of MRI systems, the signal bias caused by concomitant dephasing (Equations (4), (5), (6), (7), (8), (9), (10)) was simulated. This was done for Maxwell‐compensated waveforms after interpolation to the gradient raster time (Δ*t* = 10 μs) as well as after correction of such waveforms for the interpolation errors. We denote these waveforms “before correction” and “after correction,” respectively. For each system configuration, the diffusion encoding gradient waveforms were rotated to find the worst signal bias between rotations within the prescribed FOV. The FOV was defined as a sphere centered at the scanner isocenter with a radius of 10 cm and the signal bias was evaluated on the surface of the sphere. To determine the distribution of biases that could be observed, the waveforms were also randomly rotated 10^3^ times, and the worst signal bias was calculated on the whole sphere for each rotation. Note that the signal bias due to concomitant dephasing always results in reduced signal, and the errors are thus presented as absolute values.

### 3.5 Relative Encoding Efficiency Before and After Correction

Despite the subtle changes introduced by the correction method, the resulting gradient waveform is expected to have a slightly different encoding efficiency. To quantify this, the b‐values for each waveform were computed both before (*b*_pre_) and after correction (*b*_post_), across all combinations of gradient amplitude and magnetic field strength. The relative encoding efficiency was then calculated as (*b*_pre_ − *b*_post_)/*b*_pre_. The reduction in diffusion encoding efficiency was also assessed by quantifying the additional encoding time required to achieve the desired b‐value by extending the duration of the entire waveform until the desired b‐value was reached.

### 3.6 Simulation of Signal Error due to Concomitant Diffusion Weighting

In addition to signal errors from dephasing, the concomitant gradient introduces changes to the b‐tensor, which could yield inaccurate signal interpretation. The impact of this error was evaluated by simulating the b‐value error and the corresponding signal bias from concomitant diffusion weighting, according to Equations ((12), (13), (14), (15)). For each non‐corrected waveform, the actual b‐value was calculated for the waveform rotations that yielded the worst positive and the worst negative error. The relative b‐value error (*b*_a_ − *b*_d_)/*b*_d_ and the corresponding signal bias were reported, given that the diffusion coefficient in Equation (14) was *D* = 1 μm^2^/ms. Note that corrected waveforms are expected to express similar concomitant b‐value errors since their shape changes only subtly.

Finally, the errors due to concomitant dephasing and diffusion weighting were compared in terms of signal bias. These were visualized together as a function of *g*_max_/*B*_0_ to collapse the diffusion weighting errors onto a line (Equation 16), which allows a straightforward distinction of the dominant source of error.

### 3.7 Practical Validation

To validate the waveform correction, we performed measurements at a clinical MRI system with *g*_max_ = 200 mT/m and a maximal slew rate of *s*_max_ = 200 T/m/s (MAGNETOM Cima.X, Siemens Healthineers, Forchheim, Germany). We used a custom research pulse sequence that enabled user‐defined gradient waveforms for a diffusion‐weighted spin‐echo with EPI readout [36]. To emphasize errors caused by interpolation, asymmetric gradient waveforms for spherical b‐tensor encoding were optimized at a temporal resolution of Δ*t* = 2 ms. The waveforms had an asymmetry of 6 ms (δ_1_ − δ_2_ = 6 ms) and a total duration of 69 ms. Additionally, to demonstrate the limitations of relying solely on a stricter constraint of the Maxwell index across a broader range of MRI hardware configurations, the Maxwell index was set to 18 m^−2^ during optimization and waveform correction. Finally, to avoid flow artifacts due to convection in the oil, the waveforms were velocity‐compensated with |**m**_1_| = 0.16 s/mm before waveform correction [37], where $m_{1}=\gamma \int_{0}^{\tau }g\left(t\right)tdt$. Although velocity encoding is not explicitly controlled for in the waveform correction, it was maintained at a reduced |**m**_1_| = 0.07 s/mm. In applications where motion encoding is pivotal, a constraint on |**m**_1_| could be added in the optimizer. Measurements were performed both with waveforms before and after correction. Due to limitations from peripheral and cardiac nerve stimulation, the *g*_max_ and *s*_max_ were 166 mT/m and 63 T/m/s, respectively.

The efficacy of the waveform correction was tested in a spherical oil phantom with a diameter of 24 cm. Oil was used due to its low diffusivity, such that signal attenuation would primarily arise from dephasing caused by concomitant gradients.

To observe a wide range of concomitant gradient effects, we placed the center of the phantom at approximately *z* = 5 cm, and we used a slice thickness of 10 mm. We used axial slices with an in‐plane resolution of 2 × 2 mm^2^, FOV = 300 × 300 × 250 mm^3^, echo‐spacing = 0.64 ms, frequency encoding gradient amplitude of 24 mT/m, TR = 4000 ms, and TE = 165 ms. To minimize interference from imaging artifacts, we did not use partial‐Fourier or in/through‐plane acceleration, thereby causing a relatively long TE. The diffusion weighting was performed using asymmetric gradient waveforms at *b* = [0.0 0.1 0.5 1.0 1.5 2.0] ms/μm^2^, each executed in 10 rotations to observe the rotation dependence of the concomitant gradients. The measurement was performed using the bore coil to ensure a homogeneous image intensity across the phantom.

We investigated the diffusion‐weighted signal as a function of b‐value for all rotations of the gradient waveforms. To capture the off‐isocenter concomitant gradient behavior, the signal was extracted at **r** = [0.0 0.0 9.6] cm by manually placing a region of interest in a single slice. To isolate the effects of concomitant dephasing, that is, to remove the effects of diffusion encoding, the signal was normalized to the mean signal at each b‐value within 5 mm of the isocenter, where concomitant gradient effects are negligible. Note that this normalization removes potential effects caused by imaging gradients, thereby isolating the effects that originate from the diffusion encoding waveforms.

## 4 Results

**FIGURE 3:**
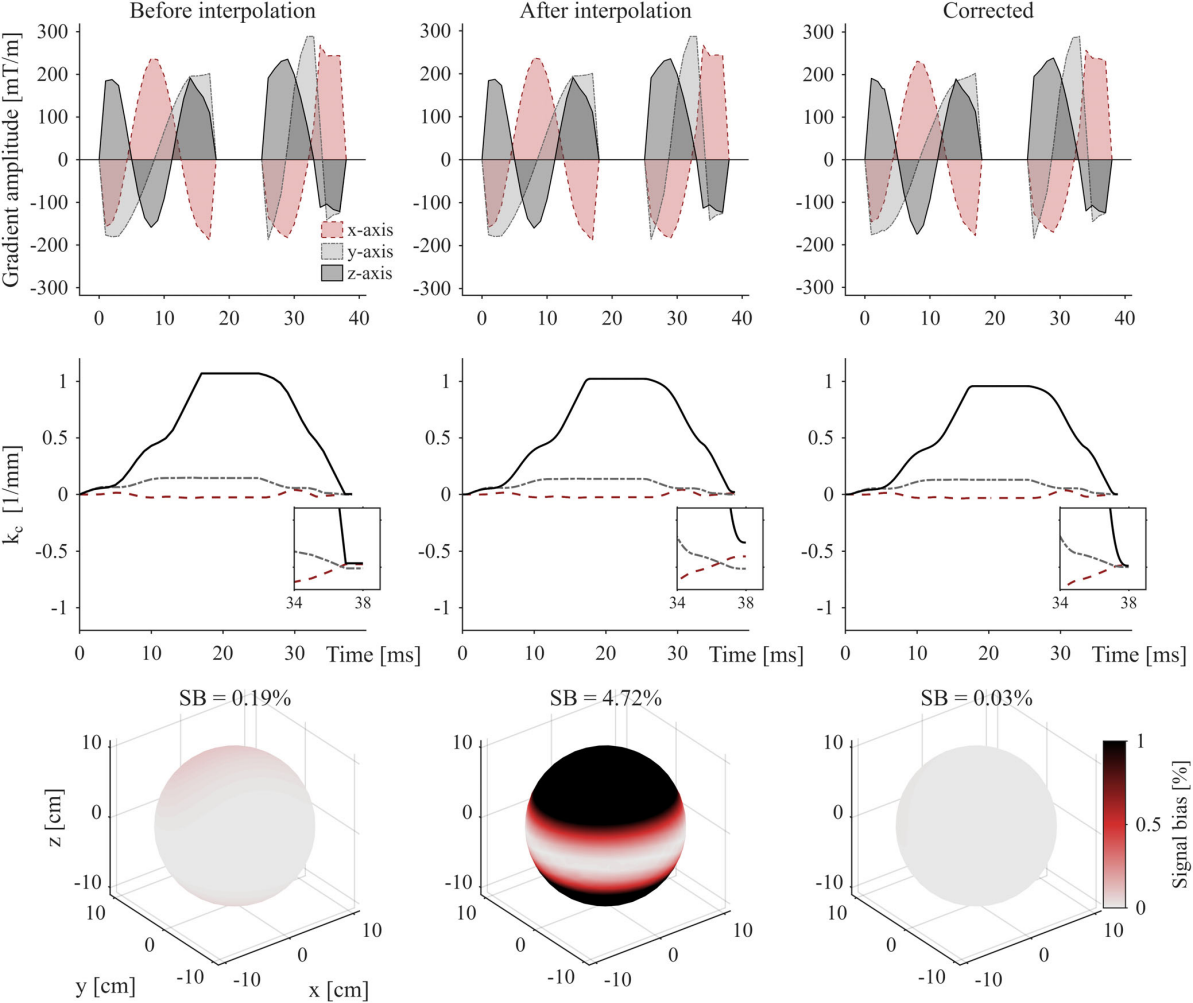
Signal bias (SB) caused by a non‐zero residual moment ($k_{c}\neq 0$, Equation 7) for non‐interpolated (left), interpolated (middle), and corrected (right) gradient waveforms. The desired gradient waveforms (top row) are optimized for a 3 T system with 300 mT/m gradients (system D). The middle row shows the concomitant dephasing vector (**q**_c_, Equation 13) of the respective gradient waveform at position **r** = [0 0 10] cm. At the end of the diffusion encoding, **k**_c_ should be equal to zero. However, a residual moment is caused by the concomitant gradients at the end of the waveform after interpolation. The signal bias is shown on the surface of a sphere with a radius of 10 cm (bottom row) using the gradient waveforms from the top row. The rotation that results in the largest bias after waveform interpolation is used consistently to illustrate the corresponding biases before interpolation and after correction. However, after waveform correction, this is not necessarily the waveform rotation producing the worst signal bias. The low temporal resolution waveform exhibits no residual dephasing and shows a maximum signal bias < 1%. However, after interpolation to the gradient raster time, a concomitant non‐zero residual moment results in a maximum signal bias of 4.72%. This bias was almost entirely removed after waveform correction (bottom right).

**FIGURE 4:**
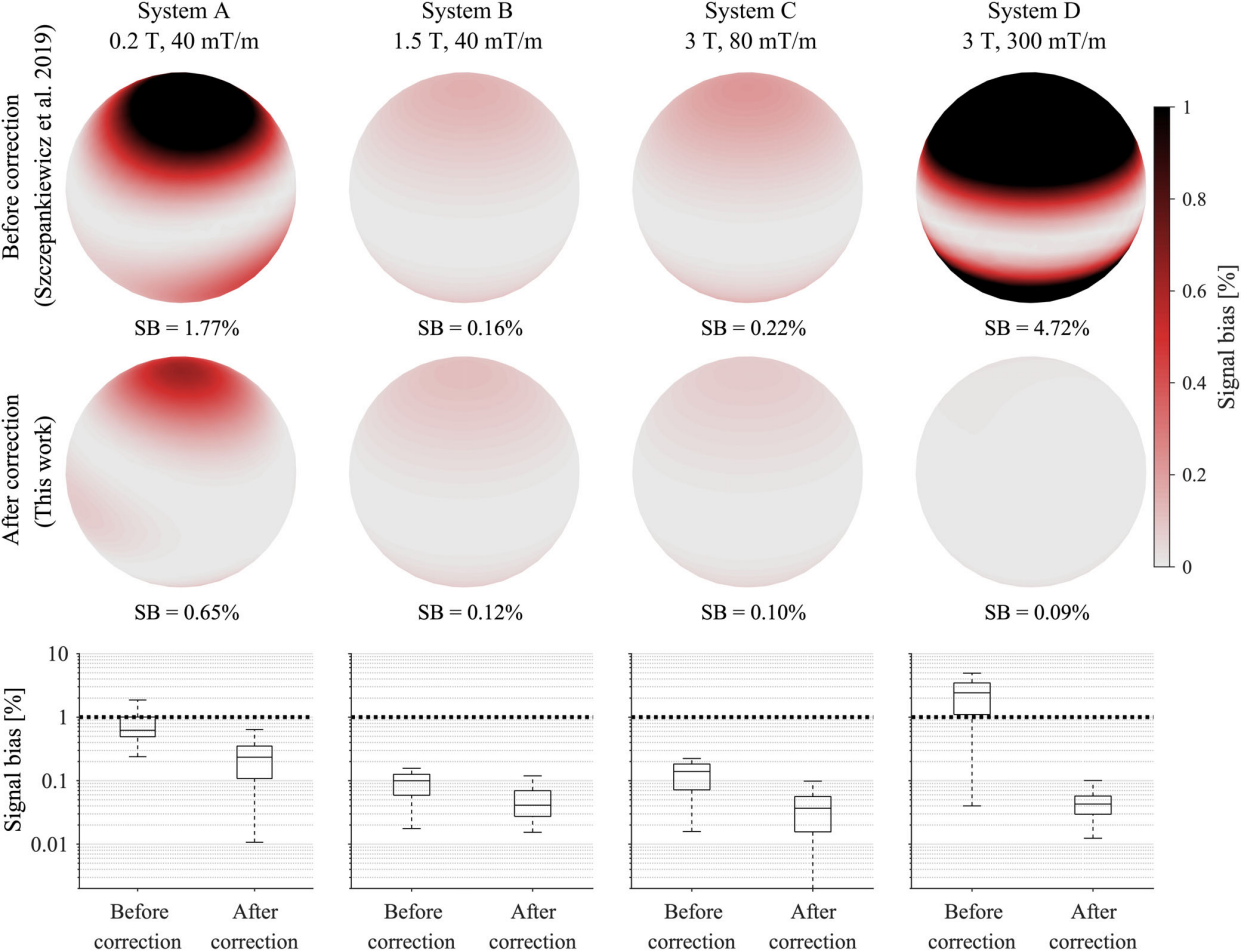
Signal bias (SB) on spheres with a radius of 10 cm for four MRI systems (A‐D). The colors show signal biases at the corresponding positions for the same coordinate frame as in Figure 3, but using the worst‐case rotation of each gradient waveform. Without additional corrections, system D exhibits substantial signal loss. However, with the proposed waveform correction, the signal bias is reduced to negligible levels (signal bias < 1%). The boxplots show the distribution of signal errors across 10^3^ different rotations of the gradient waveform. The solid line within the box indicates the median, the edges of the box represent the first and third quartiles and the whiskers extend to the 99th percentile. The correction algorithm demonstrates superior signal accuracy for all systems and rotations. Note the thick dashed line representing 1% signal bias.

**FIGURE 5:**
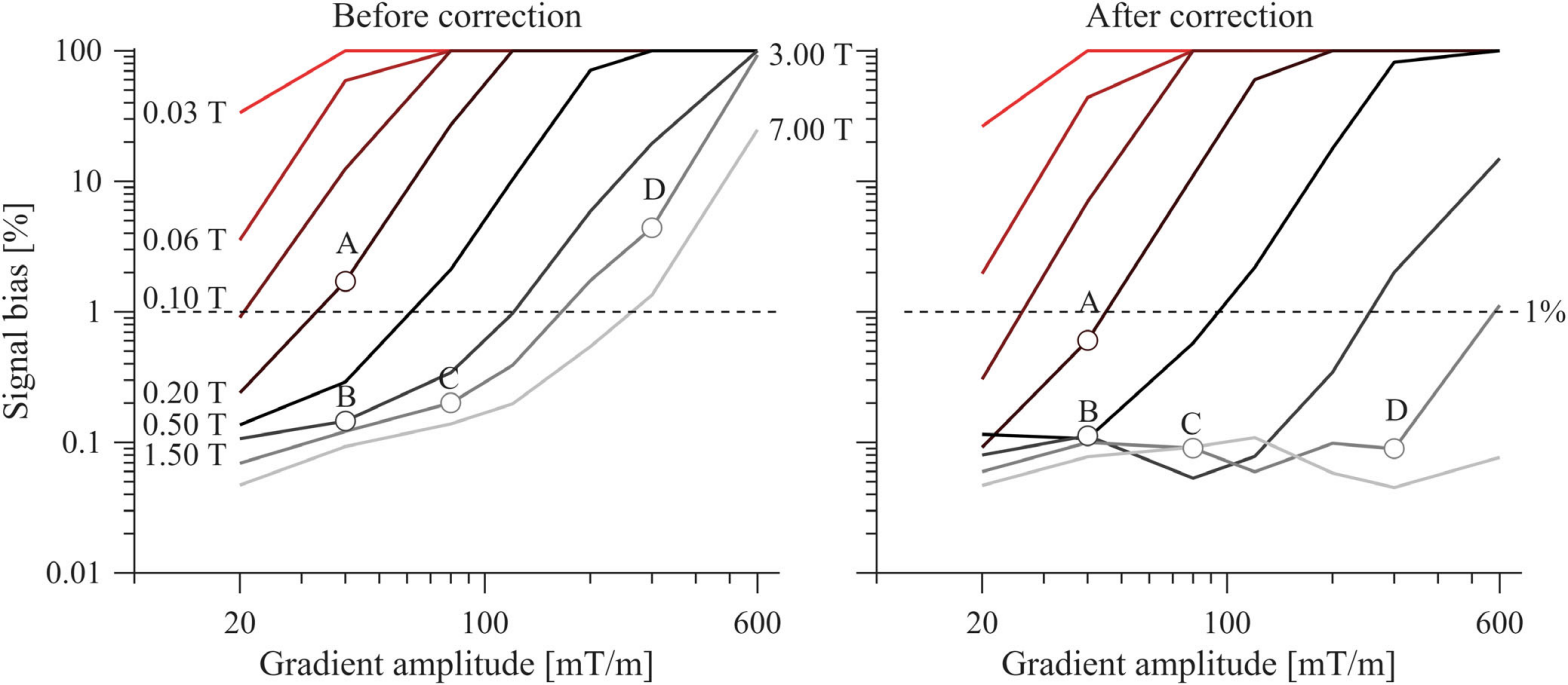
Signal bias for Maxwell‐compensated waveforms, as a function of both gradient amplitude and main magnetic field strength, before and after correction of interpolation errors. The signal bias shown is calculated for the waveform rotation resulting in the worst bias, where a slice thickness of 5 mm is assumed. Circle markers indicate the signal bias of the MRI systems characterized in Section 3.1. Signal bias increases as the gradient amplitude increases (x‐axis), and the waveform correction of interpolation errors reduces the signal bias for all feasible combinations of system parameters.

The investigation of concomitant dephasing showed that gradient waveform interpolation did not maintain Maxwell compensation. This caused gross signal errors that depended on the MRI hardware and imaging configuration (Equation 6, Figure 3). As expected, the error was negligible for the least challenging parameter combinations, that is, the 1.5 T and 40 mT/m as well as the 3 T and 80 mT/m, with a signal bias < 1%, requiring no further correction (Figure 4). However, for more challenging combinations of parameters, i.e., lower field strength and/or higher gradient amplitudes, the signal biases were above 1% (Figure 4). Indeed, several combinations exhibited a complete loss of signal somewhere in the FOV (100% bias, Figure 5).

The proposed correction method could restore Maxwell compensation and facilitate a categorical reduction of signal biases caused by concomitant dephasing. The correction takes less than 5 s per waveform when using 10 parallel active cores and 20 repetitions on an Intel Core Ultra 7155H processor. Figure 3 shows that the correction introduced a subtle modification to the desired gradient waveform, but that it markedly reduces the size of the concomitant dephasing vector. Figure 4 demonstrates the correction in four systems, where it reduced the signal bias by an order of magnitude and thereby below the 1% threshold. Notably, the correction facilitated accurate signal retrieval even for the challenging system configuration of 3 T and 300 mT/m, for which it reduced the worst‐case signal bias from 4.72% to 0.09%. However, despite its categorical improvement of signal accuracy, the correction method could not eliminate signal bias for the most extreme MRI configurations. Signal biases above 1% were observed at, for example, ultra‐low‐field *B*_0_ ≤ 0.1 T with a *g*_max_ ≥ 20 mT/m as well as ultra‐strong gradient systems with *g*_max_ > 500 mT/m and *B*_0_ ≤ 3 T (Figure 5).

**FIGURE 6:**
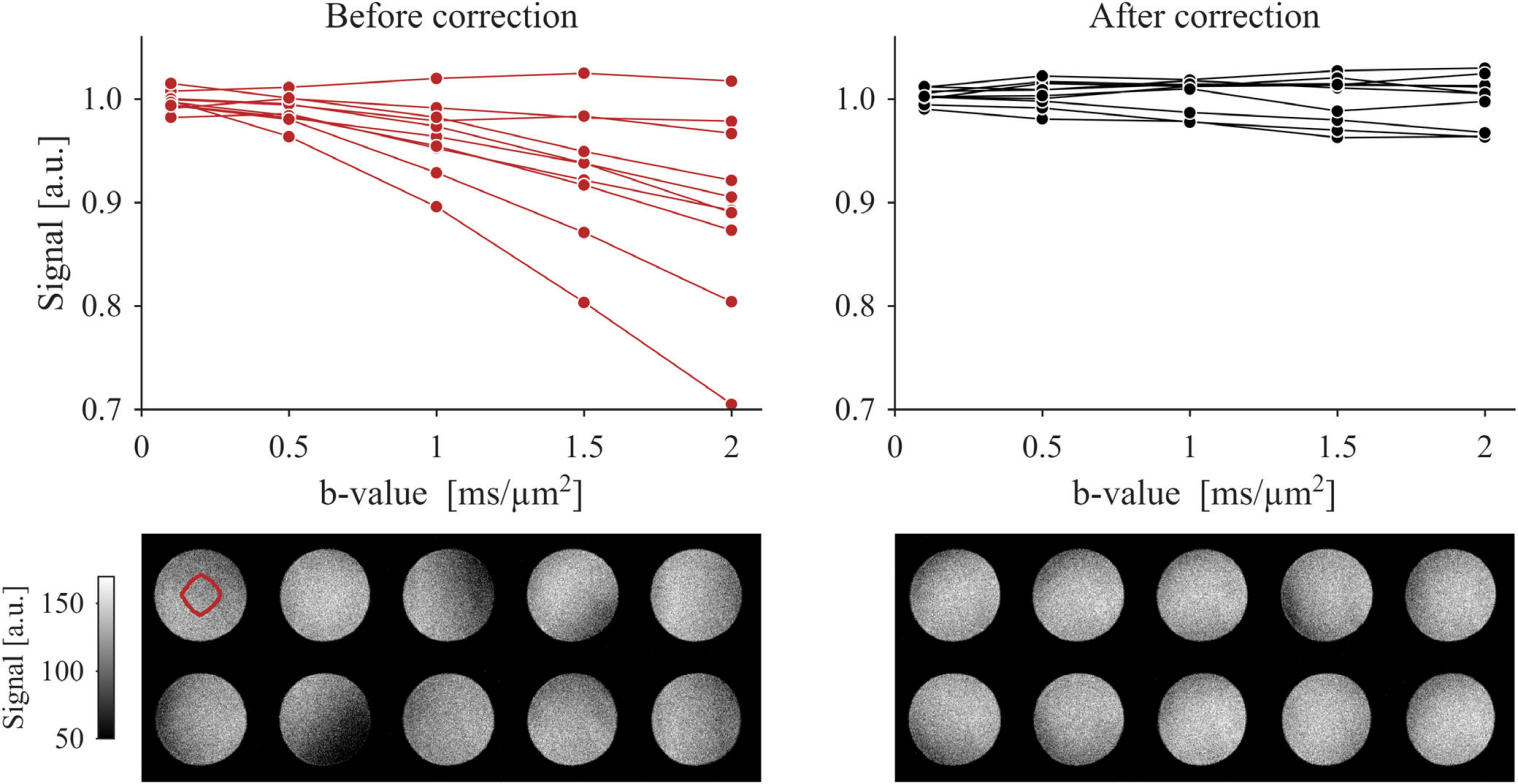
Signal versus b‐value in an oil phantom for 10 different waveform rotations (different lines and insert plots) before and after waveform correction at *z* = 9.6 cm shows that the waveforms before correction suffer from unbalanced concomitant gradients due to interpolation errors, whereas waveforms after correction recover Maxwell compensation. The signals for the waveforms before correction exhibit marked loss of signal as a function of b‐value, indicating a signal bias due to concomitant dephasing. However, the waveforms after correction yield relatively stationary signal. The bottom row shows the magnitude images of the phantom for all 10 waveform rotations at *b =* 2.0 ms/μm^2^, together with the mask used to extract the data (same mask in all images). Again, waveforms before correction yielded smoothly varying signal errors, whereas corrected waveforms yielded a more homogeneous signal with a higher similarity across rotations of the waveforms.

Practical measurements demonstrated that the waveform correction could restore Maxwell compensation to suppress concomitant dephasing and signal bias (Figure 6). By contrast, the waveforms before correction showed signal bias up to 30% in an axial slice that was 9.6 cm from the isocenter, consistent with concomitant dephasing. Note that images from the acquisitions before waveform correction exhibited a smoothly varying signal reduction across the phantom, which may be difficult to detect and attribute to concomitant gradient effects.

**FIGURE 7:**
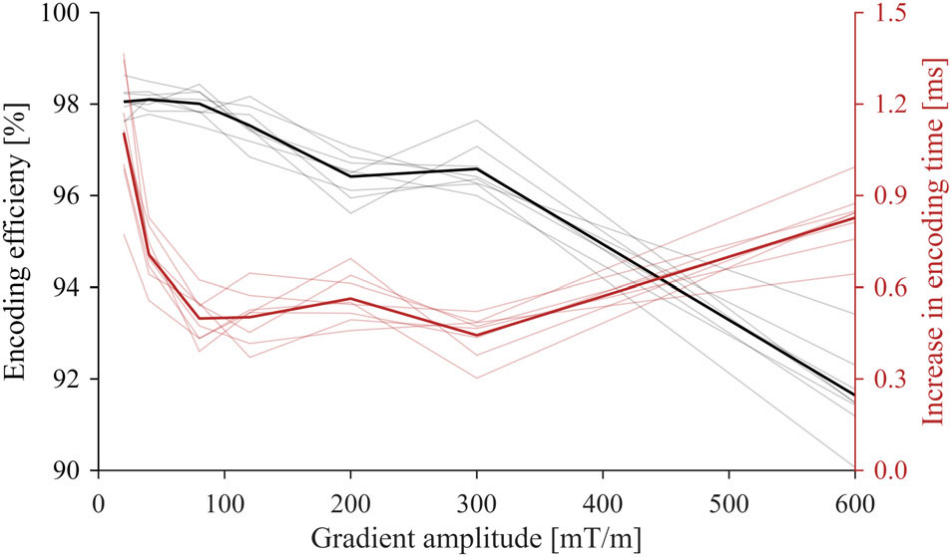
Targeting the Maxwell index in the waveform correction led to a slight reduction in encoding efficiency, resulting in a gradient encoding time increase of 0.6‐1.4 ms across all investigated hardware configurations to maintain a diffusion weighting of *b* = 2 ms/μm^2^. The left axis indicates the relative loss in encoding efficiency due to the correction algorithm, while the right axis shows the corresponding increase in encoding time. The thicker lines are the mean encoding efficiency and increase in encoding time for all *B*_0_‐*g*_max_‐combinations, whereas the thinner lines show all different waveforms. Note that encoding time can be directly interpreted as a cost in echo time. Overall, there is a consistent decrease in encoding efficiency as a function of *g*_max_. However, the resulting increase in encoding time rises at both low and high *g*_max_. This behavior reflects the realistic interplay between waveforms tailored to specific system configurations and the cost of the correction.

The waveform correction method imposed a minor reduction in encoding efficiency. At *g*_max_ < 100 mT/m, the b‐value after correction was reduced by approximately 2%, whereas at higher *g*_max_, it was reduced by up to 8% (Figure 7). To maintain the original b‐value, we estimated that the encoding time and consequently the echo time, must be extended by between 0.6 and 1.4 ms.

**FIGURE 8:**
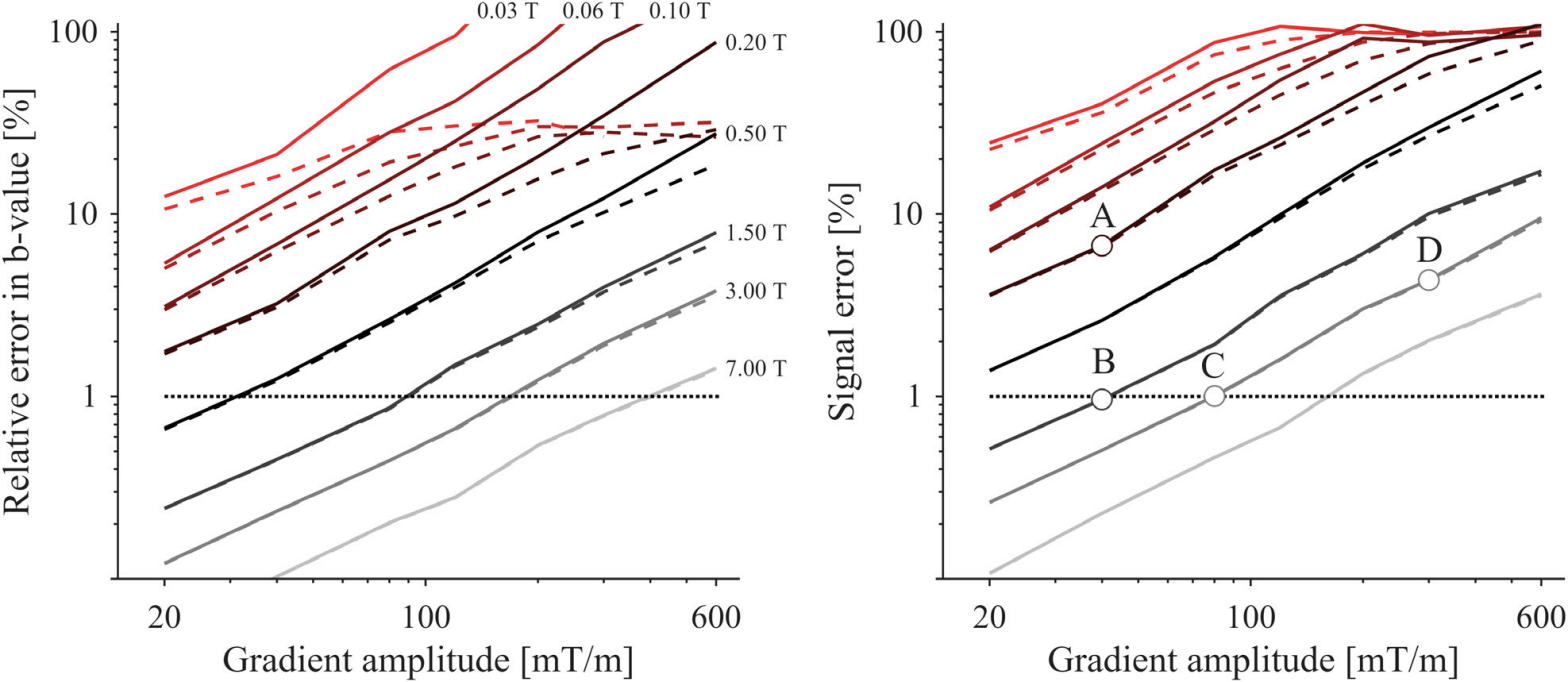
Relative error in b‐value (left) caused by the concomitant gradients for waveforms before correction as a function of *g*_max_ and *B*_0_, with the corresponding signal error (right). The solid lines show the worst positive error (increased b‐value), whereas the dashed lines show the worst negative error (decreased b‐value). With increasing b‐value error, the positive and negative biases diverge, likely because the negative error in *b* is bounded, whereas the positive error is not. Notably, clinical scanners with (B) 1.5 T/40 mT/m and (C) 3 T/80 mT/m show a signal error of 1% due to the concomitant error in b‐value. Moreover, a (A) low‐field system (*B*_0_ = 0.2 T and *g*_max_ = 40 mT/m) shows a signal error of approximately 7%, whereas the signal error for a (D) high‐performance scanner (*B*_0_ = 3 T and *g*_max_ = 300 mT/m) is 4%. The signal error due to concomitant diffusion weighting is virtually the same before and after waveform correction (data not shown), since the correction only accounts for dephasing while the overall waveform shape remains similar.

The signal bias due to concomitant diffusion weighting was observed to be a relevant consideration across a wide range of systems (Figure 8). This includes conventional MRI hardware configurations at 1.5‐3 T and 40‐80 mT/m, for which the resulting signal error was approximately 1%. At systems with lower *B*_0_ (e.g., 0.2 T and 40 mT/m) or high *g*_max_ (e.g., 3 T and 300 mT/m), the errors were larger at 4 to 7%.

**FIGURE 9:**
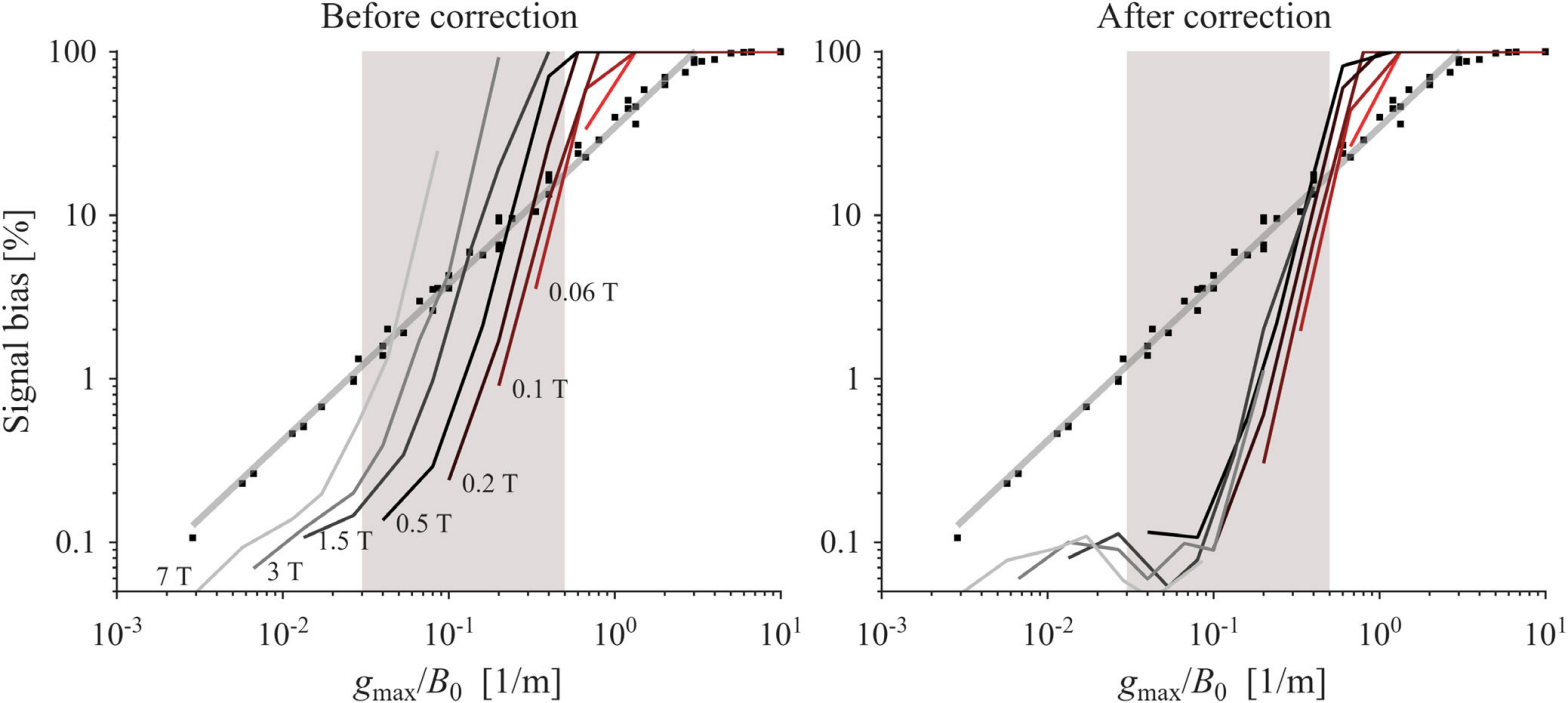
Signal bias caused by concomitant dephasing (solid lines) and concomitant diffusion weighting (black squares). The thick gray line highlights the trend of signal bias from concomitant diffusion weighting below *g*_max_/*B*_0_ = 5 m^−1^, demonstrating that they scale with *g*_max_/*B*_0_ (Equation 16). Since this collapses all systems onto one line, we omit the labels. Depending on the *B*_0_‐*g*_max_‐combination, different sources of bias dominate (Equation 11 and Equation 16). Left of the shaded region (low *g*_max_/*B*_0_), the concomitant diffusion weighting dominates the signal bias, whereas on the right (high *g*_max_/*B*_0_), the dominant source is concomitant dephasing. Inside the shaded region (intermediate *g*_max_/*B*_0_), the dominant effect depends on *g*_max_ and *B*_0_, and whether waveform correction was applied. The region where *g*_max_/*B*_0_ is between 0.05 and 0.10 m^−1^ appears to be the most advantageous for waveform correction under the simulated experimental parameters. Note that this graph varies with imaging parameters, tissue diffusivity, etc., and therefore serves mainly to highlight that the two effects do not have a simple hierarchy.

Finally, the dominant source of signal bias (concomitant dephasing vs. concomitant diffusion weighting) depends on the specific *B*_0_‐*g*_max_‐combination (Figure 9). At the low end of *g*_max_/*B*_0_ (< 0.04 m^−1^), the concomitant diffusion weighting dominates, although, the signal bias is less than 1%. At higher *g*_max_/*B*_0_ (> 0.1 m^−1^), the errors are mainly due to concomitant dephasing, reaching 100% signal loss at *g*_max_/*B*_0_ = 0.6 m^−1^, even for waveforms after correction.

## 5 Discussion

In this study, a comprehensive investigation of concomitant gradient effects across a wide range of MRI hardware configurations was conducted. As expected from theory [6], the challenges related to these effects are inflated for MRI systems with low magnetic field strengths and/or high gradient amplitudes. Notably, this investigation required derivation of an accurate and compact expression for the concomitant gradient, which includes all higher‐order contributions (Equation 4), as well as the extension of the Maxwell index to incorporate the effect of spin‐species‐specific gyromagnetic ratio and magnetic field strength (Equation 17). To separate two distinct mechanisms, we categorized the effects of concomitant gradients in terms of “concomitant dephasing” and “concomitant diffusion weighting.”

Concomitant dephasing is present when the residual moment of the actual dephasing vector (**k**_a_, Equation 7) is non‐zero at the signal readout. This is only a concern for asymmetric waveforms since symmetric waveforms have equal concomitant gradients on both sides of the refocusing pulse, which ensures that **k**_c_ = 0. To compensate for concomitant dephasing, Maxwell‐compensated gradient waveforms have been used [8]. However, despite Maxwell compensation, concomitant dephasing was present due to interpolation and higher‐order concomitant gradient terms.

We showed that the use of gradient waveform interpolation, required to adapt the gradient waveform to the gradient raster time prescribed by the hardware, has a marked impact on the Maxwell index, leading to increased concomitant dephasing and decreased signal accuracy (Figures 3, 5, and 6). This was previously identified as a potential source of error by Szczepankiewicz et al. [8], and we have corroborated this claim and investigated its relation to hardware specifications. Although it has a relatively small effect on conventional MRI systems (e.g., 1.5 T and 40 mT/m or 3 T and 80 mT/m), our investigation of a wider range of hardware configurations highlights that it can cause gross signal errors for MRI systems with lower field strength and/or stronger gradients. This is despite using Maxwell‐compensated gradient waveforms (Figures 4, 5, 6). To our knowledge, this was not previously recognized and thus motivated the development of our correction method.

The correction method proposed in this work introduces subtle changes to the desired gradient waveform to effectively achieve the desired Maxwell compensation. This, in turn, provides a categorical improvement of signal accuracy, effectively expanding the range of MRI systems for which signal can be accurately measured (Figures 5 and 6). Because of the improved signal accuracy, a more homogeneous image of the phantom is obtained. Furthermore, the waveform correction appears to be most effective in the region of *g*_max_/*B*_0_ between 0.05 and 0.10 m^−1^ (Figures 5 and 9). However, this region could change for another set of specific experimental parameters, such as slice thickness, readout method, encoding durations, etc. The key advantage of this method is that it needs to be applied only once in the design stage of a given waveform, whereafter it produces more accurate data. This also means that existing waveforms can be corrected to comply with a given MRI system, accounting for a given configuration of raster time, *B*_0_, and *g*_max_. The drawback is a relatively small penalty to diffusion encoding efficiency. For the hardware and imaging configurations investigated in the present study, the worst‐case scenario was that the encoding time was extended by 1.4 ms. Alternatively, errors that originate from interpolation could be partially avoided by evaluating the Maxwell index at a high temporal resolution during the initial waveform optimization, thereby accounting for the error introduced in the conversion from continuous to discrete waveforms. However, implementing this into the optimization framework [19] was outside the scope of this work and would not allow correction of already existing gradient waveforms.

Despite using corrected gradient waveforms with the desired Maxwell compensation (μ < μ_thr_), there were several MRI system configurations that still exhibited high signal bias above 1% due to concomitant dephasing. Current evidence suggests that this observation is the effect of higher‐order contributions to the concomitant gradients, mainly the *g* ^3^/*B*_0_^2^‐term [5, 6]. These effects could be exposed by using our complete description of the concomitant gradients (Equation 4) rather than a truncated expansion (see Supporting Information). This is notable since most investigations of concomitant fields and concomitant gradients have used an approximation that only considers leading‐order effects, i.e., those proportional to *g* ^2^/*B*_0_ (Equation 6). This premature truncation leads to inaccuracies as higher‐order effects become relevant at low *B*_0_ and high *g*_max_ [2, 5, 38, 39, 40]. Indeed, since the Maxwell index is designed to constrain only the leading *g* ^2^/*B*_0_‐term, it is expected to fail when higher‐order terms become relevant. This was observed at realistic and contemporary system configurations with strong gradients (*B*_0_ < 3 T, *g*_max_ > 300 mT/m) and at low fields (*B*_0_ < 0.1 T, *g*_max_ > 40 mT/m), for which the current method for Maxwell compensation and waveform correction was insufficient to suppress the signal bias to below 1% (Figure 5). Suppression of higher‐order contributions by modifying the Maxwell index will be investigated in future studies.

Concomitant diffusion weighting was identified to be a non‐negligible source of bias. This was observed throughout most of the hardware configurations, including conventional MRI systems with field strengths of 1.5‐3 T and gradient amplitudes of 40‐80 mT/m (Figure 8). Surprisingly, we found that the dominant source of error depends on the system configuration; concomitant diffusion weighting dominates when errors are overall low (at low *g*_max_ and high *B*_0_) but is overtaken by concomitant dephasing errors as *g*_max_/*B*_0_ is increased (Figure 9). At sufficiently extreme hardware combinations, both sources readily cause gross signal errors. Naturally, as the waveform correction is employed to suppress concomitant dephasing, the concomitant diffusion weighting does not change and is likely to become the dominant source of error.

We emphasize that, unlike concomitant dephasing, concomitant diffusion weighting is a relevant source of error independent of the gradient waveform design and voxel size, i.e., it must be considered for symmetric gradient waveform designs as well as high‐resolution and 3D readouts. However, since concomitant diffusion weighting induces a predictable error in the diffusion encoding (see Equation 4 and shared software), it can be accounted for in the analysis by performing calculations based on **b**_a_(**r**) rather than the nominal **b**_d_ (Equation 12), where **b**_a_(**r**) includes the concomitant diffusion weighting as well as imaging/readout gradients. As the actual b‐tensor varies with position, this must be done for each voxel in the FOV, similar to the correction for magnetic field non‐linearity [41]. Alternatively, the diffusion encoding could be adjusted during the measurement so that some part of the excited slice exhibits **b**_a_(**r**) = **b**_d_, however, investigating the efficacy of such an approach was outside the scope of this work. Naturally, this correction approach extends to other experimental parameters based on **g**_a_, such as motion encoding, exchange weighting, and restriction weighting.

Several limitations should be recognized in this study. First, a set of arbitrary conditions was adopted to provide a wide range of challenging but realistic scenarios. We reiterate that the actual effects of concomitant gradients will ultimately depend on factors such as gradient waveform design, FOV, voxel size, and readout method, and that the resulting signal bias should be judged in relation to other sources of error and variance. Given that not all cases are covered herein, we have shared our tools in open source to facilitate simulations of parameter combinations outside of the scope of this work (see the Data Availability Statement section). Second, the estimation of the actual gradient waveform, albeit more general and accurate than the more conventional truncated Taylor expansion, still assumes a linearly varying magnetic field (see eq. 13 in Bernstein et al. [6]), which could affect its accuracy, especially at large distances from the isocenter, where the magnetic field is likely to diverge from linearity [41]. Nevertheless, such effects can be incorporated if the gradient coil design is known beforehand [42].

## 6 Conclusions

The introduction of a wider range of magnetic field strengths and gradient amplitudes increases the challenges that originate from concomitant gradients. We have confirmed that assumptions and approaches that were sufficient for hitherto mainstream MRI hardware parameters no longer hold, and that this warrants a careful reevaluation of the design of gradient waveforms for dMRI, especially at low field strengths and high gradient amplitudes.

We showed that interpolation compromises Maxwell compensation and causes concomitant dephasing and gross signal bias. Our gradient waveform correction method restored Maxwell compensation and enabled a categorical improvement to the signal accuracy, effectively expanding the range of systems that can exploit the benefits of asymmetric diffusion encoding gradient waveforms. However, we also established that there exist realistic parameter combinations, at ultra‐low fields and ultra‐high gradients, for which the correction was still insufficient. This indicates that future correction methods must consider higher‐order contributions.

Finally, we demonstrated that signal errors caused by concomitant diffusion weighting are generally smaller than those from concomitant dephasing, but cannot be assumed to be negligible. For certain hardware configurations, they can even be the dominant source of error. Since concomitant diffusion weighting arises in both symmetric and asymmetric gradient waveform designs and can reach relevant magnitudes even at conventional MRI systems, it may be the most prevalent of the two. However, since the concomitant diffusion weighting can be predicted, the associated bias can be mitigated by accounting for it in the analysis.

## Funding

This work was supported by the Swedish Research Council (2021‐04844), the Swedish Cancer Society (220592 JIA and 222011 Pj), eSSENCE: the e‐Science Collaboration (eSSENCE@LU 12:3), the Swedish Prostate Cancer Federation, and the Crafoord Foundation (20240791).

## Conflicts of Interest

Filip Szczepankiewicz is an inventor on patents related to the subject and declares financial interests in Random Walk Imaging, AB. Frederik Testud is an employee of Siemens Healthcare AB, Sweden. Emil Ljungberg is an employee of Philips Healthcare. The other authors declare no conflicts of interest.

## Supporting information


**Figure S1:** The Taylor approximations (up to 2nd, 3rd, and 4th order) of the concomitant gradient are inaccurate at an example ultra‐low‐field system (*B*_0_ = 0.06 T and *g*_max_ = 40 mT/m). By contrast, the full analytical formula (Eq. 5) coincides perfectly with the numerical reference based on calculations of the local magnetic field. The desired gradient **g**_d_ = [0.02 0.01 0.03] was scaled to the desired gradient amplitude. The concomitant gradients (**g**_c_ = [*g*_c,x_ *g*_c,y_ *g*_c,z_]^T^) are shown as a function of positions along the diagonal where *x* = *y* = *z*.
**Figure S2:** Signal bias calculated for an ultra‐low‐field system (*B*_0_ = 0.06 T and *g*_max_ = 40 mT/m) with a slice thickness of 5 mm and |**r**| = 10 cm. The left plot shows the signal bias using the 2nd order Taylor approximation, which predicts a signal bias of 0.04%. The right shows the signal bias obtained from the full expression of the actual gradient waveform (Eq. 4), yielding 46% bias. Hence, the truncated Taylor expansion underestimates the bias. Note the different color scales in the plots.
**Figure S3:** Signal bias on the surface of a sphere with radius 10 cm centered on the isocenter as a function of coil symmetry factor α. The distributions show bias values across the surface of the sphere and across 100 random rotations of the gradient waveform. The signal bias was calculated for a 3 T system utilizing 300 mT/m gradients and a slice thickness of 5 mm, where the gradient waveform is corrected for concomitant gradient dephasing after interpolation. The maximum signal bias is approximately 0.1%, indicating that Maxwell compensation generalizes across coil symmetry factors.

## Data Availability

The numerical optimization framework used for the generation of waveforms is available in open source at https://github.com/jsjol/NOW. The waveforms used in this work, together with related resources for waveform correction, analysis of concomitant gradient effects, and data, are shared at https://github.com/filip‐szczepankiewicz/Olsson_MRM_2026_Maxwell_2, and the main functions used are available at https://github.com/filip‐szczepankiewicz/fwf_sequence_tools.

## ENDNOTES

^1^
We use the terms “field non‐linearity” and “gradient non‐uniformity,” rather than the misnomer “non‐linear gradients,” to emphasize that the ideal case would be linear fields and uniform/homogeneous gradients.

^2^
Note that the notations **q** and **k**, used in diffusion encoding and imaging, respectively, refer to the same physical quantity.

^3^
Note that the scaling is described in Szczepankiewicz et al. [22] contains a typographical error, i.e., each diagonal element of the scaling matrix should be $\sqrt{\lambda^{\prime }/\lambda }$ instead of $\lambda^{\prime }/\lambda$.

## References

[1] M. A. Bernstein , K. F. King , and X. J. Zhou , “Part III, Gradients,” in Handbook of MRI Pulse Sequences (Elsevier, 2004), 215–364, https://www.sciencedirect.com/book/monograph/9780120928613/handbook‐of‐mri‐pulse‐sequences.

[2] D. G. Norris and J. M. S. Hutchison , “Concomitant Magnetic Field Gradients and Their Effects on Imaging at Low Magnetic Field Strengths,” Magnetic Resonance Imaging 8, no. 1 (1990): 33–37. 2325514 10.1016/0730-725x(90)90209-k

[3] X. J. Zhou , S. G. Tan , and M. A. Bernstein , “Artifacts Induced by Concomitant Magnetic Field in Fast Spin‐Echo Imaging,” Magnetic Resonance in Medicine 40, no. 4 (1998): 582–591. 9771575 10.1002/mrm.1910400411

[4] Y. P. Du , X. Joe Zhou , and M. A. Bernstein , “Correction of Concomitant Magnetic Field‐Induced Image Artifacts in Nonaxial Echo‐Planar Imaging,” Magnetic Resonance in Medicine 48, no. 3 (2002): 509–515. 12210916 10.1002/mrm.10249

[5] B. de Vos , R. F. Remis , and A. G. Webb , “Characterization of Concomitant Gradient Fields and Their Effects on Image Distortions Using a Low‐Field Point‐of‐Care Halbach‐Based MRI System,” Magnetic Resonance in Medicine 91, no. 2 (2024): 828–841. 37749850 10.1002/mrm.29879

[6] M. A. Bernstein , X. J. Zhou , J. A. Polzin , et al., “Concomitant Gradient Terms in Phase Contrast MR: Analysis and Correction,” Magnetic Resonance in Medicine 39, no. 2 (1998): 300–308. 9469714 10.1002/mrm.1910390218

[7] R. Lorenz , J. Bock , J. Snyder , J. G. Korvink , B. A. Jung , and M. Markl , “Influence of Eddy Current, Maxwell and Gradient Field Corrections on 3D Flow Visualization of 3D CINE PC‐MRI Data,” Magnetic Resonance in Medicine 72, no. 1 (2014): 33–40. 24006013 10.1002/mrm.24885 PMC3872262

[8] F. Szczepankiewicz , C. F. Westin , and M. Nilsson , “Maxwell‐Compensated Design of Asymmetric Gradient Waveforms for Tensor‐Valued Diffusion Encoding,” Magnetic Resonance in Medicine 82 (2019): 1424–1437. 31148245 10.1002/mrm.27828 PMC6626569

[9] C. A. Baron , R. M. Lebel , A. H. Wilman , and C. Beaulieu , “The Effect of Concomitant Gradient Fields on Diffusion Tensor Imaging,” Magnetic Resonance in Medicine 68, no. 4 (2012): 1190–1201. 22851517 10.1002/mrm.24120

[10] C. Meier , M. Zwanger , T. Feiweier , and D. Porter , “Concomitant Field Terms for Asymmetric Gradient Coils: Consequences for Diffusion, Flow, and Echo‐Planar Imaging,” Magnetic Resonance in Medicine 60, no. 1 (2008): 128–134. 18581353 10.1002/mrm.21615

[11] E. Aliotta , K. Moulin , and D. B. Ennis , “Eddy Current‐Nulled Convex Optimized Diffusion Encoding (EN‐CODE) for Distortion‐Free Diffusion Tensor Imaging With Short Echo Times,” Magnetic Resonance in Medicine 79, no. 2 (2018): 663–672. 28444802 10.1002/mrm.26709

[12] G. Yang and J. A. McNab , “Eddy Current Nulled Constrained Optimization of Isotropic Diffusion Encoding Gradient Waveforms,” Magnetic Resonance in Medicine 81 (2018): 1818–1832. 30368913 10.1002/mrm.27539 PMC6347544

[13] J. Finsterbusch , “Double‐Spin‐Echo Diffusion Weighting With a Modified Eddy Current Adjustment,” Magnetic Resonance Imaging 28, no. 3 (2010): 434–440. 20071120 10.1016/j.mri.2009.12.004

[14] T. G. Reese , O. Heid , R. M. Weisskoff , and V. J. Wedeen , “Reduction of Eddy‐Current‐Induced Distortion in Diffusion MRI Using a Twice‐Refocused Spin Echo,” Magnetic Resonance in Medicine 49, no. 1 (2003): 177–182. 12509835 10.1002/mrm.10308

[15] E. C. Wong , R. W. Cox , and A. W. Song , “Optimized Isotropic Diffusion Weighting,” Magnetic Resonance in Medicine 34, no. 2 (1995): 139–143. 7476070 10.1002/mrm.1910340202

[16] S. Mori and P. van Zijl , “Diffusion Weighting by the Trace of the Diffusion Tensor Within a Single Scan,” Magnetic Resonance in Medicine 33, no. 1 (1995): 41–52. 7891534 10.1002/mrm.1910330107

[17] K. Butts , J. Pauly , A. de Crespigny , and M. Moseley , “Isotropic Diffusion‐Weighted and Spiral‐Navigated Interleaved EPI for Routine Imaging of Acute Stroke,” Magnetic Resonance in Medicine 38, no. 5 (1997): 741–749. 9358448 10.1002/mrm.1910380510

[18] O. Heid and J. Weber , “Diffusion Tensor Trace Pulse Sequences,” in Proceedings of 5th ISMRM (1997), 224.

[19] J. Sjölund , F. Szczepankiewicz , M. Nilsson , D. Topgaard , C. F. Westin , and H. Knutsson , “Constrained Optimization of Gradient Waveforms for Generalized Diffusion Encoding,” Journal of Magnetic Resonance 261 (2015): 157–168. 26583528 10.1016/j.jmr.2015.10.012 PMC4752208

[20] E. Aliotta , H. H. Wu , and D. B. Ennis , “Convex Optimized Diffusion Encoding (CODE) Gradient Waveforms for Minimum Echo Time and Bulk Motion‐Compensated Diffusion‐Weighted MRI,” Magnetic Resonance in Medicine 77, no. 2 (2017): 717–729. 26900872 10.1002/mrm.26166

[21] J. Finsterbusch , “Eddy‐Current Compensated Diffusion Weighting With a Single Refocusing RF Pulse,” Magnetic Resonance in Medicine 61, no. 3 (2009): 748–754. 19132755 10.1002/mrm.21899

[22] F. Szczepankiewicz , C. F. Westin , and M. Nilsson , “Gradient Waveform Design for Tensor‐Valued Encoding in Diffusion MRI,” Journal of Neuroscience Methods 348 (2021): 109007. 33242529 10.1016/j.jneumeth.2020.109007 PMC8443151

[23] M. H. Mazurek , B. A. Cahn , M. M. Yuen , et al., “Portable, Bedside, Low‐Field Magnetic Resonance Imaging for Evaluation of Intracerebral Hemorrhage,” Nature Communications 12, no. 1 (2021): 5119. 10.1038/s41467-021-25441-6 PMC8387402 34433813

[24] Y. Liu , A. T. L. Leong , Y. Zhao , et al., “A Low‐Cost and Shielding‐Free Ultra‐Low‐Field Brain MRI Scanner,” Nature Communications 12, no. 1 (2021): 7238. 10.1038/s41467-021-27317-1 PMC8671508 34907181

[25] T. O'Reilly , W. M. Teeuwisse , and A. G. Webb , “Three‐Dimensional MRI in a Homogenous 27 cm Diameter Bore Halbach Array Magnet,” Journal of Magnetic Resonance 307 (2019): 106578. 31470234 10.1016/j.jmr.2019.106578

[26] S. Biber , “MAGNETOM Free.Max: Access to MRI—How to Make It Big Inside and Small Outside,” in MAGNETOM Flash, Senior System Architect & Principal Key Expert at Siemens Healthineers, R&D AEP, 2023, 45–49.

[27] K. Setsompop , R. Kimmlingen , E. Eberlein , et al., “Pushing the Limits of In Vivo Diffusion MRI for the Human Connectome Project,” NeuroImage 80 (2013): 220–233. 23707579 10.1016/j.neuroimage.2013.05.078 PMC3725309

[28] T. K. F. Foo , E. T. Tan , M. E. Vermilyea , et al., “Highly Efficient Head‐Only Magnetic Field Insert Gradient Coil for Achieving Simultaneous High Gradient Amplitude and Slew Rate at 3.0 T (MAGNUS) for Brain Microstructure Imaging,” Magnetic Resonance in Medicine 83, no. 6 (2020): 2356–2369. 31763726 10.1002/mrm.28087

[29] S. Y. Huang , T. Witzel , B. Keil , et al., “Connectome 2.0: Developing the Next‐Generation Ultra‐High Gradient Strength Human MRI Scanner for Bridging Studies of the Micro‐, Meso‐ and Macro‐Connectome,” NeuroImage 243 (2021): 118530. 34464739 10.1016/j.neuroimage.2021.118530 PMC8863543

[30] D. A. Feinberg , A. J. S. Beckett , A. T. Vu , et al., “Next‐Generation MRI Scanner Designed for Ultra‐High‐Resolution Human Brain Imaging at 7 Tesla,” Nature Methods 20, no. 12 (2023): 2048–2057. 38012321 10.1038/s41592-023-02068-7 PMC10703687

[31] M. Weiger , J. Overweg , M. B. Rösler , et al., “A High‐Performance Gradient Insert for Rapid and Short‐T2 Imaging at Full Duty Cycle,” Magnetic Resonance in Medicine 79, no. 6 (2018): 3256–3266. 28983969 10.1002/mrm.26954

[32] X. J. Zhou , Y. P. du , M. A. Bernstein , H. G. Reynolds , J. K. Maier , and J. A. Polzin , “Concomitant Magnetic‐Field‐Induced Artifacts in Axial Echo Planar Imaging,” Magnetic Resonance in Medicine 39, no. 4 (1998): 596–605. 9543422 10.1002/mrm.1910390413

[33] F. Testud , D. Gallichan , K. J. Layton , et al., “Single‐Shot Imaging With Higher‐Dimensional Encoding Using Magnetic Field Monitoring and Concomitant Field Correction,” Magnetic Resonance in Medicine 73, no. 3 (2015): 1340–1357. 24687529 10.1002/mrm.25235

[34] C. F. Westin , H. Knutsson , O. Pasternak , et al., “Q‐Space Trajectory Imaging for Multidimensional Diffusion MRI of the Human Brain,” NeuroImage 135 (2016): 345–362. 26923372 10.1016/j.neuroimage.2016.02.039 PMC4916005

[35] F. Szczepankiewicz , C. Eichner , A. Anwander , C. F. Westin , and M. Paquette , “The Impact of Gradient Non‐Linearity on Maxwell Compensation When Using Asymmetric Gradient Waveforms for Tensor‐Valued Diffusion Encoding,” in Proceedings of the International Society for Magnetic Resonance in Medicine, 2020, 1–4.

[36] F. Szczepankiewicz , J. Sjölund , F. Ståhlberg , J. Lätt , and M. Nilsson , “Tensor‐Valued Diffusion Encoding for Diffusional Variance Decomposition (DIVIDE): Technical Feasibility in Clinical MRI Systems,” PLoS One 14, no. 3 (2019): e0214238. 30921381 10.1371/journal.pone.0214238 PMC6438503

[37] F. Szczepankiewicz , J. Sjölund , E. Dall'Armellina , et al., “Motion‐Compensated Gradient Waveforms for Tensor‐Valued Diffusion Encoding by Constrained Numerical Optimization,” Magnetic Resonance in Medicine 85, no. 4 (2021): 2117–2126. 33048401 10.1002/mrm.28551 PMC7821235

[38] D. A. Yablonskiy , A. L. Sukstanskii , and J. J. Ackerman , “Image Artifacts in Very Low Magnetic Field MRI: The Role of Concomitant Gradients,” Journal of Magnetic Resonance 174, no. 2 (2005): 279–286. 15862245 10.1016/j.jmr.2005.02.016 PMC2140255

[39] W. R. Myers , M. Mossle , and J. Clarke , “Correction of Concomitant Gradient Artifacts in Experimental Microtesla MRI,” Journal of Magnetic Resonance 177, no. 2 (2005): 274–284. 16169266 10.1016/j.jmr.2005.08.003

[40] J. O. Nieminen and R. J. Ilmoniemi , “Solving the Problem of Concomitant Gradients in Ultra‐Low‐Field MRI,” Journal of Magnetic Resonance 207, no. 2 (2010): 213–219. 20884262 10.1016/j.jmr.2010.09.001

[41] R. Bammer , M. Markl , A. Barnett , et al., “Analysis and Generalized Correction of the Effect of Spatial Gradient Field Distortions in Diffusion‐Weighted Imaging,” Magnetic Resonance in Medicine 50, no. 3 (2003): 560–569. 12939764 10.1002/mrm.10545

[42] N. G. Lee , S. Popescu , D. Grodzki , A. Krug , S. X. Cui , and K. S. Nayak , “General Expression of Concomitant Gradient Terms Including Gradient Nonlinearity for Higher Order Image Reconstruction,” in ISMRM Workshop on Data Sampling and Image Reconstruction 2026.

