# Supplementary material for: Concomitant Gradient Effects Across Field Strengths and Gradient Amplitudes: Improved Estimation of Errors and Correction of Concomitant Dephasing and Diffusion Weighting
Source: Magn Reson Med. 2026 Jun 11;96(3):1178–91. doi: 10.1002/mrm.70422 (PMC13327446; doi:10.1002/mrm.70422)
Supplement: Supplementary file 1 — Figure S1: The Taylor approximations (up to 2nd, 3rd, and 4th order) of the concomitant gradient are inaccurate at an example ultra‐low‐field system (B 0 = 0.06 T and g max = 40 mT/m). By contrast, the full analytical formula (Eq. 5) coincides perfectly with the numerical reference based on calculations of the local magnetic field. The desired gradient g d = [0.02 0.01 0.03] was scaled to the desired gradient amplitude. The concomitant gradients (g c = [g c,x g c,y g c,z]T) are shown as a function of positions along the diagonal where x = y = z. Figure S2: Signal bias calculated for an ultra‐low‐field system (B 0 = 0.06 T and g max = 40 mT/m) with a slice thickness of 5 mm and |r| = 10 cm. The left plot shows the signal bias using the 2nd order Taylor approximation, which predicts a signal bias of 0.04%. The right shows the signal bias obtained from the full expression of the actual gradient waveform (Eq. 4), yielding 46% bias. Hence, the truncated Taylor expansion underestimates the bias. Note the different color scales in the plots. Figure S3: Signal bias on the surface of a sphere with radius 10 cm centered on the isocenter as a function of coil symmetry factor α. The distributions show bias values across the surface of the sphere and across 100 random rotations of the gradient waveform. The signal bias was calculated for a 3 T system utilizing 300 mT/m gradients and a slice thickness of 5 mm, where the gradient waveform is corrected for concomitant gradient dephasing after interpolation. The maximum signal bias is approximately 0.1%, indicating that Maxwell compensation generalizes across coil symmetry factors. [file MRM-96-1178-s001.docx]

# Supplementary Materials

The following expression for the actual gradient waveform ($\mathbf{g}_{a}=\mathbf{g}+\mathbf{g}_{c}$) was derived through symbolic differentiation of equation A10 in the work by Bernstein et al. [1], using the symbolic toolbox in Matlab (2024b, The MathWorks, Natick, MA, USA). Note that, to improve legibility, we write the desired gradient waveform without the subscript ‘d’, and we refrain from writing out its dependence on time ($\mathbf{g}=\mathbf{g}_{d}(t)=[g_{x} g_{y} g_{z}]$).

The initial result from symbolic differentiation was manually simplified to state $\mathbf{g}_{a}$ at a given position ($\mathbf{r}=[x, y, z]$), according to

$$\mathbf{g}_{a}=\frac{\mathbf{g}\left( B_{0}+\mathbf{g}\cdot\mathbf{r} \right)+\left[ \begin{matrix} -\alpha g_{z}\left( -\alpha g_{z}x+\beta y+g_{x}z \right)+\beta\left( \beta x+\left( \alpha-1 \right)g_{z}y+g_{y}z \right) \\ \beta\left( -\alpha g_{z}x+\beta y+g_{x}z \right)+\left( \alpha-1 \right)g_{z} \left( \beta x+\left( \alpha-1 \right)g_{z}y+g_{y}z \right) \\ g_{x}(-\alpha g_{z}x+\beta y+g_{x}z)+g_{y}(\beta x+(\alpha-1)g_{z}y+g_{y}z) \end{matrix} \right]}{\sqrt{\left( B_{0}+\mathbf{g}\cdot\mathbf{r} \right)^{2}+\left( -\alpha g_{z}x+\beta y+g_{x}z \right)^{2}+\left( \beta x+\left( \alpha-1 \right)g_{z}y+g_{y}z \right)^{2}}} ,$$

which was further simplified, by using the substitution $\mathbf{v}=\left[ -\alpha g_{z}, \beta, g_{x} \right]$ and $\mathbf{w}=\left[ \beta, \left( 1-\alpha\right)g_{z}, g_{y} \right]$, to yield the final compact expression (Eq. 4)

$$\mathbf{g}_{a}=\frac{\mathbf{g}B_{0}\boldsymbol{+}\left( \mathbf{g}^{\otimes2}+\mathbf{v}^{\otimes2}+\mathbf{w}^{\otimes2} \right)\cdot\mathbf{r}}{\sqrt{\left( B_{0}+\boldsymbol{g\cdot r} \right)^{2}+\left( \boldsymbol{v\cdot r} \right)^{\mathbf{2}}\mathbf{+}\left( \boldsymbol{w\cdot r} \right)^{\mathbf{2}}}}=\frac{\mathbf{g}B_{d}\boldsymbol{+}\left( \mathbf{v}^{\otimes2}+\mathbf{w}^{\otimes2} \right)\cdot\mathbf{r}}{\sqrt{B_{d}^{2}+\left( \boldsymbol{v\cdot r} \right)^{\mathbf{2}}\mathbf{+}\left( \boldsymbol{w\cdot r} \right)^{\mathbf{2}}}}=\frac{\mathbf{J}^{T}\mathbf{B}_{a}}{\left| \mathbf{B}_{a} \right|}$$

where $B_{d}=B_{0}+\boldsymbol{g\cdot r}$, $\otimes$ denotes the outer product such that $\mathbf{g}^{\otimes2}=\mathbf{g}\otimes\mathbf{g}$. The right hand side shows that the expression is related to the Jacobian (**J**, first-order spatial derivative) of **B**_a_ [1].

These functions are available in the open-source repository <https://github.com/filip-szczepankiewicz/fwf_sequence_tools>. The full concomitant gradient (Eq. 4) is calculated by the function fwf_gwf_to_gwf_actual and an approximation of the truncated Taylor expansion for a given order is calculated by fwf_gwf_to_gwf_actual_taylorOrder [1].

Eq. 4 was validated by comparing it to numerical differentiation of the local magnetic field strength described by Eq. 1. Figure S1 shows that Eq. 4 is consistent with the numerical calculation of the gradient, whereas the Taylor expansions show limited accuracy.

The value of a more accurate description of concomitant gradients can be seen in Figure S2, where we visualize the signal bias predicted by the 2^nd^ order Taylor approximation and the full expression for the actual gradient waveform (Eq. 4). The figure shows that the truncated approximation predicts negligible errors, whereas, the more accurate expression (Eq. 4) reveals that the actual errors are large.

Finally, we show that Maxwell compensation also works for asymmetric gradient coils wherein, i.e., for any asymmetry factor α in the interval 0 to 1 (Eq. 1). Figure S3 shows a stationary and low signal bias as function of *α*, indicating that Maxwell compensation generalizes across coil configurations [2].

| 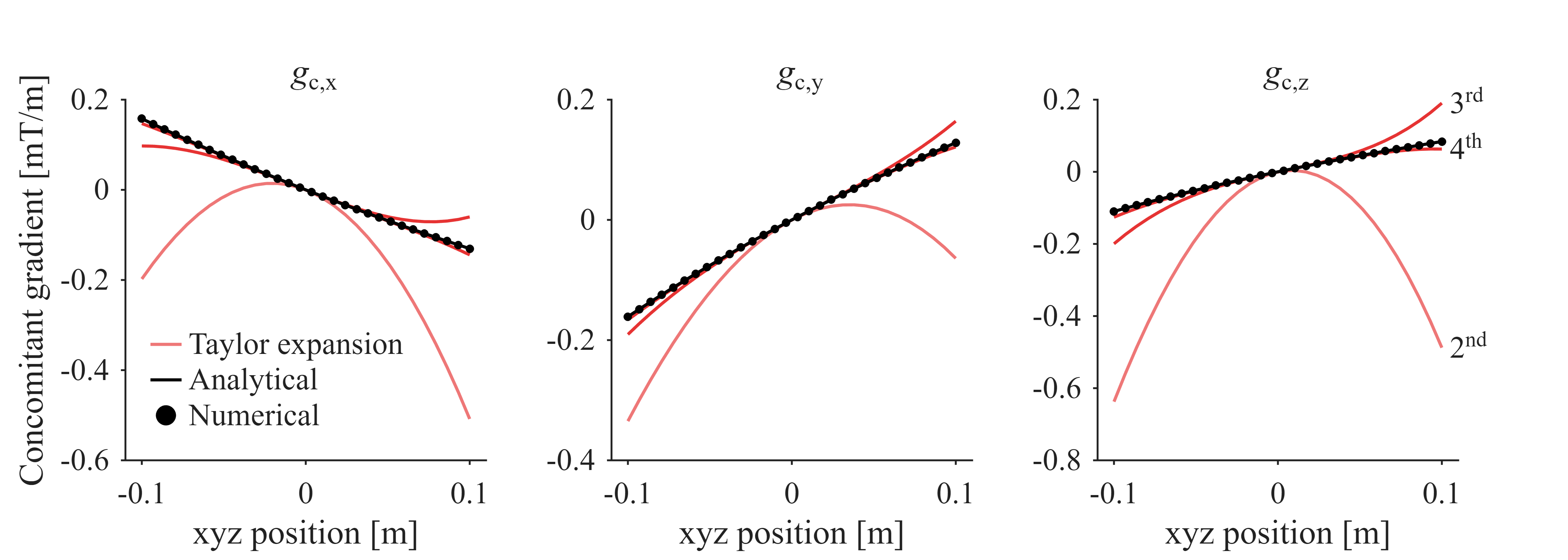  Figure S1 – The Taylor approximations (up to 2^nd^, 3^rd^, and 4^th^ order) of the concomitant gradient are inaccurate at an example ultra-low-field system (*B*_0_ = 0.06 T and *g*_max_ = 40 mT/m). By contrast, the full analytical formula (Eq. 5) coincides perfectly with the numerical reference based on calculations of the local magnetic field. The desired gradient **g**_d_ = [0.02 0.01 0.03] was scaled to the desired gradient amplitude. The concomitant gradients (**g**_c_ = [*g*_c,x_ *g*_c,y_ *g*_c,z_]^T^) are shown as a function of positions along the diagonal where *x* = *y* = *z*. |
| --- |

| 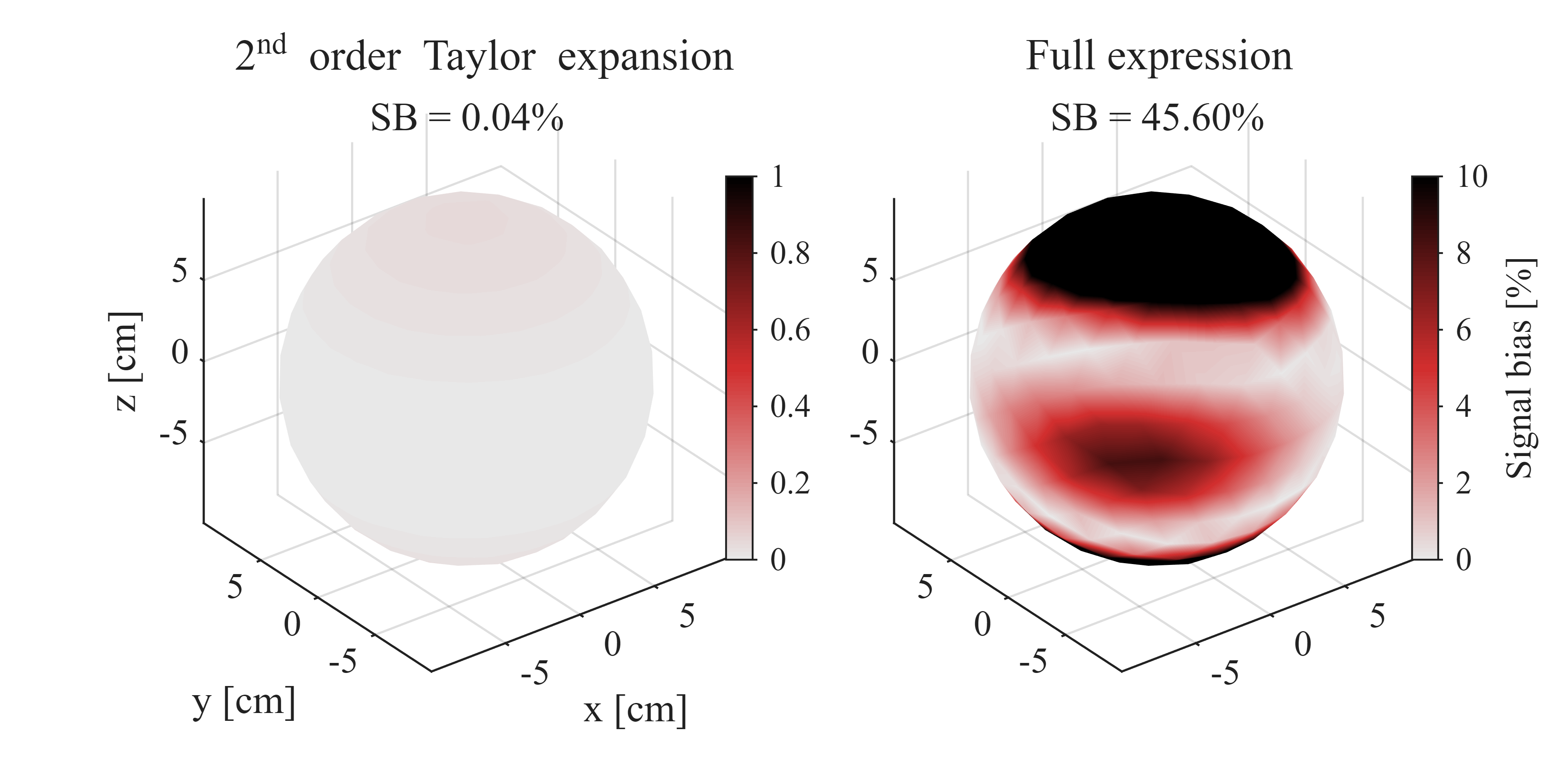  Figure S2 – Signal bias calculated for an ultra-low-field system (*B*_0_ = 0.06 T and *g*_max_ = 40 mT/m) with a slice thickness of 5 mm and \|**r**\| = 10 cm. The left plot shows the signal bias using the 2^nd^ order Taylor approximation, which predicts a signal bias of 0.04%. The right shows the signal bias obtained from the full expression of the actual gradient waveform (Eq. 4), yielding 46% bias. Hence, the truncated Taylor expansion underestimates the bias. Note the different color scales in the plots. |
| --- |

| 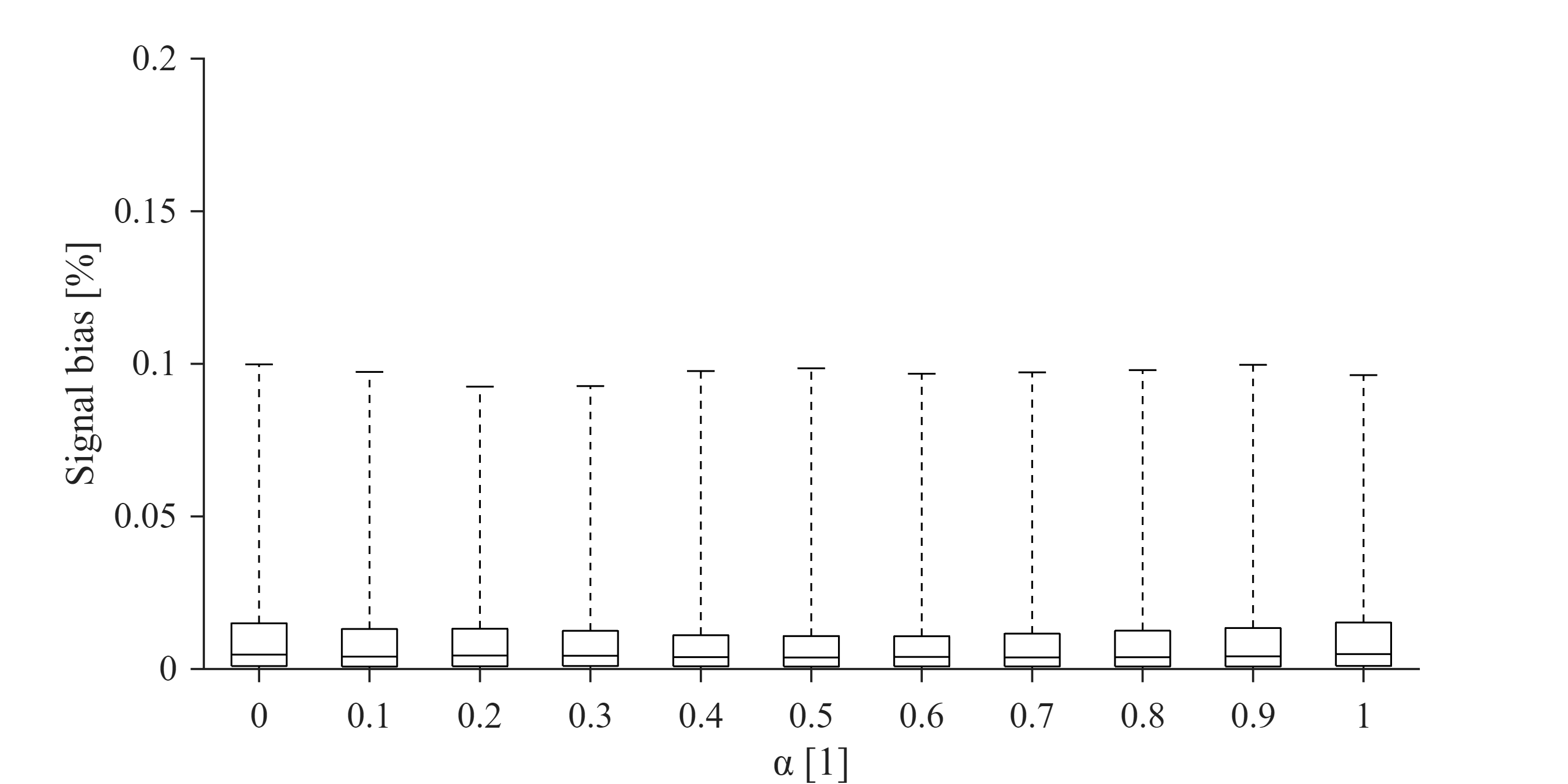  Figure S3 – Signal bias on the surface of a sphere with radius 10 cm centered on the isocenter as a function of coil symmetry factor *α*. The distributions show bias values across the surface of the sphere and across 100 random rotations of the gradient waveform. The signal bias was calculated for a 3 T system utilizing 300 mT/m gradients and a slice thickness of 5 mm, where the gradient waveform is corrected for concomitant gradient dephasing after interpolation. The maximum signal bias is approximately 0.1%, indicating that Maxwell compensation generalizes across coil symmetry factors. |
| --- |

# References

1. Bernstein, M.A., et al., *Concomitant gradient terms in phase contrast MR: analysis and correction.* Magn Reson Med, 1998. **39**(2): p. 300–8.

2. Szczepankiewicz, F., et al. *The impact of gradient non-linearity on Maxwell compensation when using asymmetric gradient waveforms for tensor-valued diffusion encoding*. in *Proc. Intl. Soc. Mag. Reson. Med. 28*. 2020. Virtual.
